# Behavioral and Contextual Patterns Derived From Ecological Momentary Assessment and Wearable Sensors and Their Associations With Health-Related Outcomes: WEALTH Cross-Sectional Study

**DOI:** 10.2196/85991

**Published:** 2026-08-11

**Authors:** Junko Kose, Jerome Bouchan, Léopold K Fezeu, Nastasia Gauffeny, Christoph Buck, Richard Cimler, Alan Donnelly, Steriani Elavsky, Janas Harrington, Grainne Hayes, Antje Hebestreit, Luis Sigcha, Tomas Vetrovsky, Greet Cardon, Jean-Michel Oppert

**Affiliations:** 1 Nutritional Epidemiology Research Team (EREN), Université Sorbonne Paris Nord and Université Paris Cité, INSERM, INRAE, CNAM, Center of Research in Epidemiology and StatisticS (CRESS), 74 rue Marcel Cachin, Bobigny, 93017, France, 33 148388930; 2 Leibniz Institute for Prevention Research and Epidemiology - BIPS, Bremen, Germany; 3 Faculty of Science, University of Hradec Kralove, Hradec Kralove, Czech Republic; 4 Department of Physical Education and Sport Sciences, University of Limerick, Limerick, Ireland; 5 Health Research Institute, University of Limerick, Limerick, Ireland; 6 Physical Activity for Health Research Center, University of Limerick, Limerick, Ireland; 7 Department of Human Movement Studies, Faculty of Education, University of Ostrava, Ostrava, Czech Republic; 8 HRB Centre for Health and Diet Research, School of Public Health, University College Cork, Cork, Ireland; 9 Faculty of Physical Education and Sport, Charles University, Prague, Czech Republic; 10 Department of Movement and Sports Sciences, Faculty of Medicine and Health Sciences, Ghent University, Ghent, Belgium; 11 Department of Nutrition, Pitié-Salpêtrière Hospital (AP-HP), Sorbonne Université, Paris, France

**Keywords:** accelerometer, eating behavior, ecological momentary assessment, health-related outcomes, physical activity, sedentary behavior, sleep

## Abstract

**Background:**

Physical activity, sedentary behavior, sleep, and eating behavior are recognized as key contributors to physical and mental health. Ecological momentary assessment (EMA) can capture both the temporal variations and contextual correlates of these behaviors.

**Objective:**

This study aimed to identify, in adults, (1) multibehavioral clusters, including the social, physical, and psychological contexts as assessed by EMA; and (2) the associations of these behaviors with selected health-related outcomes.

**Methods:**

A sample of 510 participants from Czechia, Germany, France, and Ireland, with cross-sectional data collected in 2023‐2024, was included (WEALTH study; median age 35.5, IQR 25.0-50.0 y; n=289, 56.7% female participants). During a 7-day free-living period, data on sedentary behavior (activPAL), physical activity (ActiGraph), sleep, eating behavior, and contextual characteristics (EMA) were collected. Dietary intake (Food Frequency Questionnaire) was assessed prior to the 7-day period. We used factor analysis of mixed data on 35 variables and then applied hierarchical clustering on principal components. Linear regression models with robust variance were used to examine associations between the clusters and health-related quality of life (36-Item Short Form Health Survey), well-being (5-item World Health Organization Well-Being Index), and handgrip strength. A multinomial logistic regression was used to examine the association between clusters and self-rated health.

**Results:**

Four multibehavioral clusters were identified: unhealthy behavior (n=109, reference group), mixed behavior–healthy diet (n=173), mixed behavior–stable good mood (n=142), and healthy behavior (n=86). The mixed behavior–stable good mood and healthy behavior clusters showed significantly higher average scores on mental health-related quality of life (β=6.32, 95% CI 3.73‐8.91 and β=5.61, 95% CI 2.47‐8.75, respectively) and well-being (β=11.62, 95% CI 7.86‐15.38 and β=9.63, 95% CI 5.17‐14.10, respectively) compared with the unhealthy behavior cluster. Participants in these clusters and those in the mixed behavior–healthy diet cluster tended to report better perceived health than those in the unhealthy behavior cluster (odds ratio [OR] 3.19, 95% CI 1.20‐8.49; OR 9.85, 95% CI 3.54‐27.40; OR 12.18, 95% CI 4.04‐36.71, respectively).

**Conclusions:**

These findings indicate that engaging in multiple healthy lifestyle behaviors is associated with better health outcomes than focusing on a single domain and demonstrate the opportunity to identify meaningful multibehavioral patterns related to health by using EMA combined with body-worn movement sensors.

## Introduction

Physical inactivity and unhealthy diets are among the most prevalent risk factors for ill health and impaired quality of life, making them key targets for health promotion and preventive strategies [1]. Unhealthy lifestyle behaviors tend to co-occur within individuals rather than being randomly distributed across the population [2]. The combination of unfavorable lifestyle behaviors, including insufficient physical activity (PA), prolonged sedentary behavior (SB), poor diets, and inadequate sleep, may exert a greater impact on health outcomes than the sum of each behavior in isolation [3,4]. Recent systematic reviews indicate that research describing such multibehavioral patterns, or clusters, and their associations with health outcomes has predominantly focused on children and adolescents [5,6]. In adults, studies have largely focused on PA, SB, and/or eating behavior, with limited consideration of sleep [5]. Thus, to date, relatively few studies in adults have comprehensively addressed PA, SB, sleep, and eating behaviors together [5,6].

Physical behaviors, used here as a generic term to denote PA, SB, and sleep together with eating behaviors, such as dietary intake, number of meals, and meal timings, occur in daily life at different times, frequencies, and temporal sequences [7]. Moreover, physical and eating behaviors may be shaped by contextual characteristics, which themselves follow their own patterns of temporal variation [8-10]. For example, evidence suggests that sharing meals with family members or engaging regularly in PA in green spaces is associated with improved mental health outcomes [11,12]. In addition, psychological contexts (eg, affect or mood) have been suggested to have bidirectional associations, particularly with PA. Specifically, individuals experiencing negative mood states may be less motivated or inclined to engage in PA, which, in turn, can contribute to a further decline in mood and affect [13]. Therefore, taking into account contextual variables such as physical, social, and psychological contexts appears to be much needed to refine our understanding of the temporal patterning of physical and eating behaviors [7]. In the only comprehensive scoping review we found on this topic [7], among 10 included studies that applied clustering analyses to these behaviors with contexts, the studied contextual characteristics (including physical, social, and psychological contexts, among others) varied widely. In addition, none of these studies examined both physical and eating behaviors together with their respective contexts over time [7].

Among advanced methods for investigating multiple behaviors over time, together with related contextual characteristics, ecological momentary assessment (EMA) offers unique opportunities to collect large amounts of time-stamped and context-sensitive data on behaviors of interest [10,14]. The EMA methodology involves the administration of brief questionnaires to participants via a dedicated smartphone app, and can be combined with body-worn movement sensors [15]. EMA questionnaires are triggered at random at specific time intervals (time-based triggers), in response to predefined events detected through passive sensing (eg, sensor-based triggers by accelerometers), and/or upon self-initiation by the participants (self-initiated triggers) [10,14,16]. This approach transcends traditional methods of studying behaviors in isolation, facilitating the identification of high-risk populations and target contexts for prevention strategies regarding physical and eating behaviors.

The aim of this study was to provide a holistic understanding of how physical and eating behaviors, along with their respective contexts, interact in real-world settings among adults, and to explore their association with selected health-related outcomes. This was achieved by leveraging data collected using EMA and body-worn sensors in a sample of European adults. The specific objectives were (1) to identify multibehavioral clusters, including physical and eating behaviors and their physical, social, and psychological contexts, using advanced analytical techniques (ie, clustering algorithms); and (2) to explore how the multibehavioral clusters are associated with health-related outcomes such as health-related quality of life (HRQOL), well-being, self-rated health, and handgrip strength.

## Methods

### Study Design

**Figure 1.**
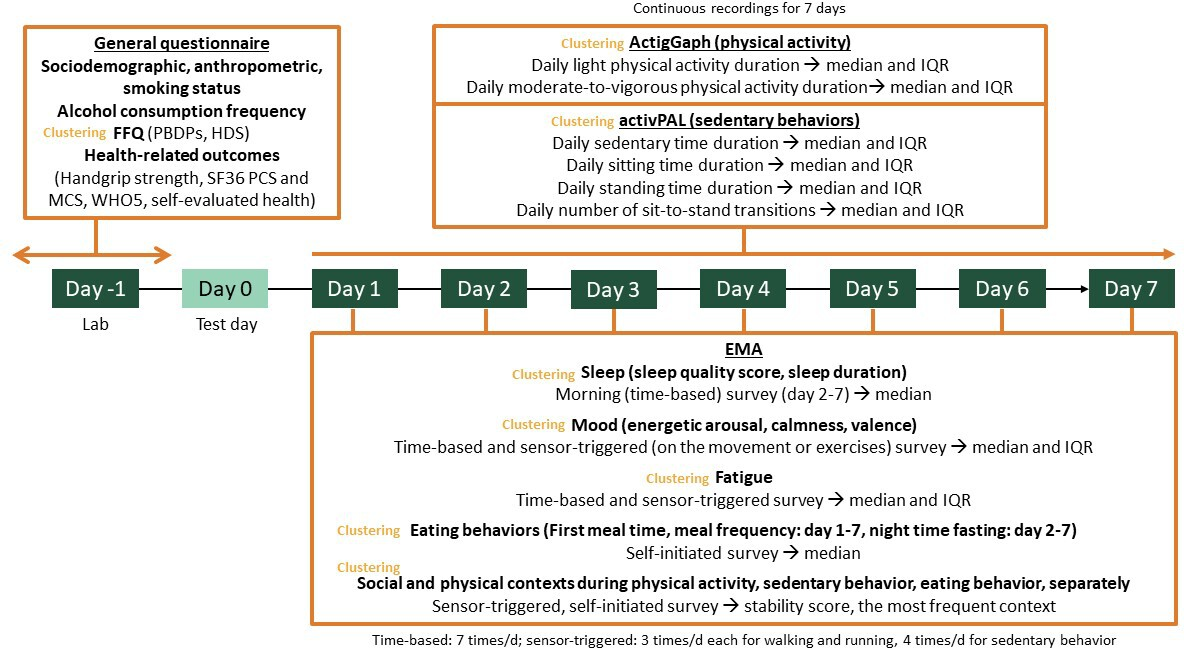
Overview of the measurement protocol; the European Wearable Sensor Assessment of Physical and Eating Behaviors (WEALTH) project. EMA: ecological momentary assessment; FFQ: Food Frequency Questionnaire; HDS: healthy diet score; MCS: mental composite score; PBDP: plant-based diet propensity score; PCS: physical composite score; SF36: 36-Item Short Form Health Survey; WHO5: 5-item World Health Organization Well-Being Index.

This study is part of the European Wearable Sensor Assessment of Physical and Eating Behaviors (WEALTH) project, which is supported by the Joint Programming Initiative Healthy Diet Healthy Life of the European Commission. The study’s design and methods have been described in detail elsewhere [17]. Briefly, participants from Czechia, Germany, France, and Ireland were recruited between March 2023 and March 2024. While participants from Czechia, Germany, and Ireland were enrolled via convenience sampling methods, such as letters, emails, telephone calls, posters, flyers, and social media, the French participants were drawn from the ongoing NutriNet-Santé cohort study, which was initiated in 2009 [18]. Participants were adults aged 18‐64 years; exclusion criteria included chronic diseases, physical impairments, and shift work. Enrollment aimed for a balanced sex split. Following a lab-based calibration session (day -1) and a test day (day 0), a 7-day free-living data collection period (day 1-7) using EMA and wearable devices formed the core of this study. In total, 627 adults completed the study. The measurement schedule is presented in Figure 1. Ethical approval was obtained in all participating countries, and informed consent was collected from all participants (see the “Ethical Considerations” section). This study builds on previous pilot and feasibility phases of the WEALTH project, which tested compliance with repeated EMA surveys, evaluated the performance of sensor-based triggers, and optimized the number and timing of prompts for large-scale data collection [16]. These earlier steps ensured that the EMA procedures applied here were feasible, acceptable, and methodologically robust.

### EMA Assessment

EMA was conducted using the HealthReact app (v1.1.17), paired with a Fitbit Charge 5 (Fitbit LLC), worn continuously on the nondominant wrist during data collection [16,19]. In addition to survey delivery, the HealthReact platform enabled the integration of time-based, sensor-triggered, and self-initiated EMA with wearable sensor data, supporting the comprehensive capture of daily behaviors and their contexts. Participants without a compatible smartphone or mobile data plan received a project-issued Android smartphone, while others used their own smartphone (iOS or Android). At the laboratory visit (day –1), participants were trained in using the EMA app, and technical issues were resolved. The EMA protocol combined time-based, sensor-triggered, and self-initiated prompts. Time-based surveys were randomly triggered 7 times daily within predefined windows (morning survey: 8-09:45 AM; daily surveys: 10:15-11:45 AM, 12:15-1:45 PM, 2:15-3:45 PM, 4:15-5:45 PM, 6:15-7:45 PM; evening survey: 8:15-9:45 PM), spaced at least 30 minutes apart. Sensor-triggered surveys were based on step cadence and heart rate data from the Fitbit Charge 5, synced with the HealthReact server automatically every 15 minutes or manually. Sedentary surveys were triggered after 20 minutes of 0 steps with heart rate detection (maximum 4 per day, ≥90 min apart). Walking surveys were triggered after 5 minutes at 60 to 139 steps/min, and running surveys after 5 minutes at 140 steps/min or more (maximum 3 per day each). For both time-based and sensor-triggered surveys, participants had 15 minutes to respond, with reminders issued after 5, 10, and 12 minutes. Eating behavior was reported via self-initiated surveys, with participants instructed to record all instances of meals, snacks, and drinks (except pure water).

EMA variables used for clustering included sleep, fatigue, mood, eating behavior, and the social and physical contexts of eating, PA, and SB. Sleep was assessed via daily sleep duration and self-rated sleep quality (“How would you rate your sleep quality last night?”; visual analog scale, 0=“very bad” to 100=“very good”) in the morning survey. Fatigue was self-rated in time-based and sensor-triggered surveys (“How would you rate your fatigue level right now?”; visual analog scale, 0=“no fatigue” to 100=“extreme fatigue”). Mood was assessed in time-based and sensor-triggered surveys across 3 dimensions (valence, calmness, and energetic arousal) using a visual analog scale (0‐100; higher scores indicating higher levels). In sensor-triggered surveys, mood was assessed only during movement, and/or PA [20]. Eating behavior indicators included daily meal frequency, first meal timing, and night-time fasting duration. Meal frequency was calculated as the total number of meals (breakfast, lunch, dinner, snacks, and beverages, except for pure water) reported per day. Night-time fasting was defined as the interval between the last meal of the previous day and the first meal of the subsequent day. Social contexts (ie, alone; with family and/or children; with friends, classmates, and/or colleagues; with strangers, crowd, organized group, and/or other; multiple companies) and physical contexts (ie, at home; at college or work; in a green space; on the move or street; other indoor locations; other outdoor locations) were combined, yielding 30 modalities for eating behavior, and similarly for PA and SB in sensor-triggered surveys.

### Physical Behavior Measurements

Participants wore 2 research-grade accelerometers: the ActiGraph wGT3X-BT (ActiGraph LLC) for PA, attached to the right hip using an elastic belt, and the activPAL 3 micro (PAL Technologies Ltd) for SB, attached to the right thigh, as per manufacturer’s guidelines [21]. Both devices were worn continuously, except during aquatic activities; in addition, the ActiGraph was removed at bedtime and while showering. Data from the ActiGraph were processed using the *GGIR* package in R (R Foundation for Statistical Computing) [22], while activPAL data were analyzed using the classification rules extraction algorithm [23,24]. A valid day was defined as 8 hours or more of wear time for the ActiGraph [25] and 20 hours or more for the activPAL. For clustering, ActiGraph variables included daily time (in minutes) spent in light PA (LPA) and moderate-to-vigorous PA (MVPA). Intensity classification was based on the median absolute deviation metric, corresponding to light (22.5-94), moderate (94-396), and vigorous PA (396+), respectively [26]. activPAL variables included the daily number of sit-to-stand transitions and daily time spent (in minutes) standing, sitting, and in total sedentary time (including sitting, seated transport, and lying, excluding sleep).

### Dietary Intake Assessment

Frequency of food and beverage intake was assessed before (Czechia and France), during (Ireland), or after (Germany) the laboratory visit (day –1) using an online 62-item Food Frequency Questionnaire (FFQ) adapted from a tool that was validated and tested for reproducibility in different European countries [27]. For each item, participants reported intake frequency over the past 4 weeks from 7 options: never, 1 to 3 times/week, 4 to 6 times/week, once/day, twice/day, 3 times/day, and 4 or more times/day. From weekly intake frequencies, the following diet scores were calculated for clustering analyses: the healthy diet score (HDS) [28], the overall plant-based diet propensity (PBDP) score, and separate healthy and unhealthy PBDP scores [29]. The HDS, based on a review of predefined diet quality scores [30], reflects adherence to dietary guidelines and includes five components: (1) sugar, (2) fat, (3) whole meal, (4) fruits and vegetables, and (5) fish. Each component is scored from 0 to 10, with a total score ranging from 0 to 50; higher scores indicate better adherence to dietary recommendations [28]. Overall PBDP was calculated as the proportion of intake from plant-based food groups (healthy and unhealthy) relative to all plant- and animal-based food groups: healthy and unhealthy PBDP scores were calculated similarly, with the numerator restricted to the respective plant-based food group [29]. Higher scores indicate more frequent intake within each category, whereas lower overall PBDP scores indicate a greater reliance on animal-based foods. In general, healthy plant-based foods include low-processed plant-based foods and/or whole grains, while unhealthy plant-based foods include those with added sugar, fat, and salt and/or refined grains (Table S1 in Multimedia Appendix 1).

### Sociodemographic and Health Measurements

Sociodemographic information was collected at baseline and included sex (male, female), age (continuous), educational level according to the International Standard Classification of Education (ISCED) [31] (<high school degree, =high school degree, >high school degree, and other), occupation (employed, unemployed, student, and others), smoking status (current smoker, ex-smoker, and nonsmoker), and alcohol consumption frequency (never, 1‐3 times/week, and ≥4 times/week). Anthropometric measurements were taken during the laboratory-based calibration session (day −1) in light clothing without shoes by trained survey staff. Height (nearest 0.25 cm) was measured with a portable stadiometer (Seca Ltd), and weight (nearest 0.1 kg) with electronic scales (Seca Ltd; TANITA EUROPE B.V.; and MyWeigh). BMI (kg/m²) was calculated as weight divided by height squared. Handgrip strength (kg) was measured twice on each hand using a digital dynamometer with adjustable grip (Takei Scientiﬁc Instruments Co. Ltd), and the maximum measurement for each participant was used.

The baseline questionnaire also included the 36-Item Short Form Health Survey (SF-36) [32] to assess HRQOL and the 5-item World Health Organization Well-Being Index (WHO-5) [33] to assess well-being. The SF-36 comprises 8 HRQOL dimensions: general health (6 items), vitality (4 items), bodily pain (2 items), physical role limitation (4 items), emotional role limitation (3 items), mental health (5 items), physical functioning (10 items), and social functioning (2 items). Two SF-36 composite scores were calculated: the mental composite score (MCS; vitality, social functioning, emotional role, and mental health), and the physical composite score (PCS; physical functioning, physical role, bodily pain, and general health), both ranging from 0 (low HRQOL) to 100 (high HRQOL) [32]. We used the specific question “In general, would you say that your health is (excellent, very good, good, fair, or poor)’’, implemented in SF-36, as an overall indicator for physical and mental self-rated health. The WHO-5 score assessed positive feelings over the past 2 weeks using 6 response options (0‐5 points); the total raw score (0-25) was multiplied by 4 to yield a 0‐100 well-being score [33]. Health-related outcomes selected for this study were the SF-36 MCS and PCS, WHO-5 score, self-rated health, and handgrip strength.

### Statistical Analyses

In a preliminary stage, multilevel regression models with between- and within-participant random intercepts were fitted for variables with repeated assessments (ie, sleep, PA, SB, eating behavior, and mood-fatigue scores). The statistical significance of the variability was determined based on *P* values obtained by comparing the between-participant random intercept model with the between- and within-participant random intercepts model, and vice-versa. Based on these results, median values per participant across time were calculated for all quantitative variables to capture between-participant variability, and the IQR per participant across time was computed for variables showing significant within-participant variability. For categorical variables describing combined social and physical contexts (combined 30 modalities, described above), the most frequent modality was identified. In addition, contextual stability scores for each participant and behavior (ie, PA, SB, and eating behavior) were calculated as negative entropy: −1*entropy ($-\sum_{i=1}^{n}pi\;log\;pi$, where *pi* represents the probability of each modality) [9]. Since entropy measures variability, multiplying it by −1 provides an indicator of stability. In this study, we applied this score to physical and social contexts to determine whether participants engaged in eating and physical behaviors within stable or variable contexts (eg, consistently eating alone vs varying between being alone, with family, or with friends). Higher scores indicate greater contextual stability [9].

Hierarchical clustering on principal components (HCPC) was performed using the *FactoMineR* package v.2.11 [34], based on components extracted from factor analysis for mixed data (FAMD). FAMD summarized the main variation patterns in datasets containing both quantitative and categorical variables, generating uncorrelated principal components that retained relevant information while reducing noise [35]. The number of components was selected according to interpretability, supported by the scree plot of eigenvalues, and the mean squared error of prediction (MSEP) obtained through k-fold cross-validation [36]. The selected components captured variations across all input categories (ie, sleep, PA, SB, eating behavior, and contextual characteristics) while ensuring optimal explained variance (inertia) and reduced prediction error. For HCPC, Euclidean distance and the Ward method were used to construct a dendrogram minimizing within-cluster inertia. The number of clusters was determined based on the gain in inertia and their interpretability. We conducted bootstrap resampling with 500 iterations and computed the Jaccard coefficient for models with 3, 4, and 5 clusters using the *fpc* R package v.2.2‐13 to evaluate the stability of the clusters [37,38]. Subsequently, k-means consolidation was applied to enhance internal cohesion within clusters. Cluster characteristics were described using the v-test from the *FactoMine*R package, which compares the means or percentages of variables within each cluster against the overall sample (higher v-values indicating greater representation in the cluster) [34]. Sociodemographic, anthropometric, smoking status, and alcohol consumption frequency were compared across clusters using the chi-squared test (or Fisher exact test, where appropriate) for categorical variables and the Kruskal-Wallis rank-sum test for continuous variables.

Associations between multibehavioral clusters and selected health-related outcomes were examined using linear regression models with robust variance estimation [39] for continuous outcomes (SF-36 MCS and PCS scores, WHO-5 score, and handgrip strength) and a multinomial logistic regression model for the categorical outcome of self-rated health (reference category: poor, fair, or good). All models were adjusted for sex, age, country, BMI, smoking status, alcohol consumption frequency, educational level, and occupation. Linear regression with robust variance was selected following the evaluation of linearity, residual distribution, and heteroscedasticity. Interaction terms (clusters by age category [≥ median and < median] and clusters by sex) were tested to assess moderation by age and sex. All data management and statistical analyses were conducted in R version 4.5.0 [40]. The significance was set at an *α* level of .05.

### Ethical Considerations

Ethics committee approval was granted in each of the 4 study centers prior to the commencement of the study. At the University of Limerick, the ethics committee approval was granted by the Education and Health Sciences Faculty Research Ethics Committee (approval number: 22_09_10_EHS_); in Bremen, approval was granted by the Ethics Committee of the University of Bremen (approval number: 2022‐25); in the Czech Republic, approval was granted by the Committee for Research Ethics at the University of Hradec Kralove (approval number: 11/2022); and in France by Comité de Protection des Personnes CPP Ile-de-France VI (approval number: 2022-A02208-35). All participants received detailed information about the purpose, procedures, risks, and benefits of the study prior to participation. Written informed consent was obtained from all participants before data collection commenced. Participants were informed of their right to withdraw at any time without penalty. Participants’ privacy and confidentiality were strictly protected. Identifying details (including names, initials, or other personal identifiers) have been omitted. A small cash reward incentive of €30-€40 (average exchange rate was approximately US $1.08 per Euro between March 2023 and March 2024) per participant was given in the Czech Republic, Germany, and Ireland to increase response rates, ensure participant compliance, and facilitate the return of all devices.

## Results

### Characteristics of Participants

Of the 627 participants who completed the study protocol, 116 with at least 1 missing clustering variable and 1 participant with missing occupation data were excluded, resulting in a final analytical sample of 510 (Figure S1 in Multimedia Appendix 1). Sociodemographic characteristics, health status, physical and eating behaviors, and contextual characteristics were generally similar between included (n=510) and excluded (n=117) participants (Tables S2 and S3 in Multimedia Appendix 1). In the final sample of 510 participants, 56.7% (n=289) were female, and the median age was 35.5 (IQR 25.0‐50.0) years. About half (n=233, 45.7%) had an educational level above high school, and 62.2% (n=317) were employed. Country representation was 23.1% (n=118), 26.5% (n=135), 25.9% (n=132), and 24.5% (n=125) from Czechia, France, Germany, and Ireland, respectively (Table 1).

**Table 1.** Characteristics of participants according to multibehavioral clusters in the European Wearable Sensor Assessment of Physical and Eating Behaviours (WEALTH) project (N=510).

Characteristic	Overall (N=510)	Unhealthy behavior (n=109)	Mixed behavior–healthy diet (n=173)	Mixed behavior–stable good mood (n=142)	Healthy behavior (n=86)	*P* value
Sex, n (%)						<.001^a^
Female	289 (56.7)	66 (60.0)	117 (67.6)	59 (41.5)	47 (54.7)	
Male	221 (43.3)	43 (39.4)	56 (32.4)	83 (58.5)	39 (45.3)	
Country, n (%)						<.001^a^
Czechia	118 (23.1)	32 (29.4)	24 (13.9)	27 (19.0)	35 (40.7)	
France	135 (26.5)	7 (6.4)	58 (33.5)	51 (35.9)	19 (22.1)	
Germany	132 (25.9)	19 (17.4)	72 (41.6)	28 (19.7)	13 (15.1)	
Ireland	125 (24.5)	51 (46.8)	19 (11.0)	36 (25.4)	19 (22.1)	
Age (y), median (IQR)	35.5 (25.0-50.0)	22.0 (20.0-35.0)	35.0 (27.0-46.0)	44.0 (30.0-57.0)	45.0 (31.0-59.0)	<.001^b^
Educational level, n (%)						<.001^a^
<High school degree	107 (21.0)	50 (45.9)	19 (11.0)	19 (13.4)	19 (22.1)	
High school degree	136 (26.7)	24 (22.0)	40 (23.1)	36 (25.4)	36 (41.9)	
>High school degree	233 (45.7)	33 (30.3)	100 (57.8)	77 (54.2)	23 (26.7)	
Others	34 (6.7)	2 (1.8)	14 (8.1)	10 (7.0)	8 (9.3)	
Occupation, n (%)						<.001^c^
Student	156 (30.6)	66 (60.0)	44 (25.4)	31 (21.8)	15 (17.4)	
Employed	317 (62.2)	40 (36.7)	119 (68.8)	100 (70.4)	58 (67.4)	
Nonemployed	17 (3.3)	2 (1.8)	7 (4.0)	3 (2.1)	5 (5.8)	
Others	20 (3.9)	1 (0.9)	3 (1.7)	8 (5.6)	8 (9.3)	
BMI (kg/m²), median (IQR)	24.0 (21.6-26.6)	23.7 (21.4-25.9)	23.5 (21.2-25.8)	24.6 (21.7-26.8)	24.6 (22.7-27.8)	.01^b^
Smoking status, n (%)						.07^a^
Current smoker	35 (6.9)	11 (10.1)	10 (5.8)	7 (4.9)	7 (8.1)	
Ex-smoker	89 (17.5)	12 (11.0)	31 (17.9)	23 (16.2)	23 (26.7)	
Nonsmoker	386 (75.7)	86 (78.9)	132 (76.3)	112 (78.9)	56 (65.1)	
Alcohol consumption, n (%)						.81^a^
≥4 times per week	44 (8.6)	10 (9.2)	15 (8.7)	11 (7.7)	8 (9.3)	
1‐3 times per week	195 (38.2)	35 (32.1)	67 (38.7)	56 (39.4)	37 (43.0)	
0 times per week	271 (53.1)	64 (58.7)	91 (52.6)	75 (52.8)	41 (47.7)	
SF-36^d^ PCS^e^, median (IQR)	56.0 (52.9-58.6)	56.5 (53.4-59.1)	56.2 (52.1-59.7)	55.7 (53.3-57.5)	56.0 (52.6-58.7)	.32^b^
SF-36 MCS^f^, median (IQR)	50.1 (41.4-54.5)	44.6 (36.2-50.8)	47.6 (37.0-52.0)	53.4 (49.9-56.8)	52.2 (46.8-56.9)	<.001^b^
WHO-5^g^ score, median (IQR)	64.0 (52.0-76.0)	56.0 (48.0-68.0)	60.0 (48.0-72.0)	72.0 (64.0-80.0)	68.0 (56.0-80.0)	<.001^b^
Handgrip strength (kg), median (IQR)	32.5 (26.9-43.0)	32.0 (27.0-43.3)	30.7 (24.4-37.5)	36.2 (28.1-45.8)	33.8 (27.1-44.7)	<.001^b^
Self-rated health, n (%)						<.001^a^
Poor, fair, or good	184 (36.1)	52 (47.7)	72 (41.6)	34 (23.9)	26 (30.2)	
Very good	238 (46.7)	49 (45.0)	71 (41.0)	79 (55.6)	39 (45.3)	
Excellent	88 (17.3)	8 (7.3)	30 (17.3)	29 (20.4)	21 (24.4)	

a Pearson chi-square test across multibehavioral patterns.

b Kruskal-Wallis rank sum test across multibehavioral patterns.

c Fisher exact test using the Monte-Carlo approach with simulated *P* value (1e+05 replicates) across multibehavioral patterns.

d SF-36: 36-Item Short Form Health Survey.

e PCS: physical composite score.

f MCS: mental composite score.

g WHO-5: 5-item World Health Organization Well-Being Index.

### Clustering Analyses: Multibehavioral Clusters

**Figure 2.**
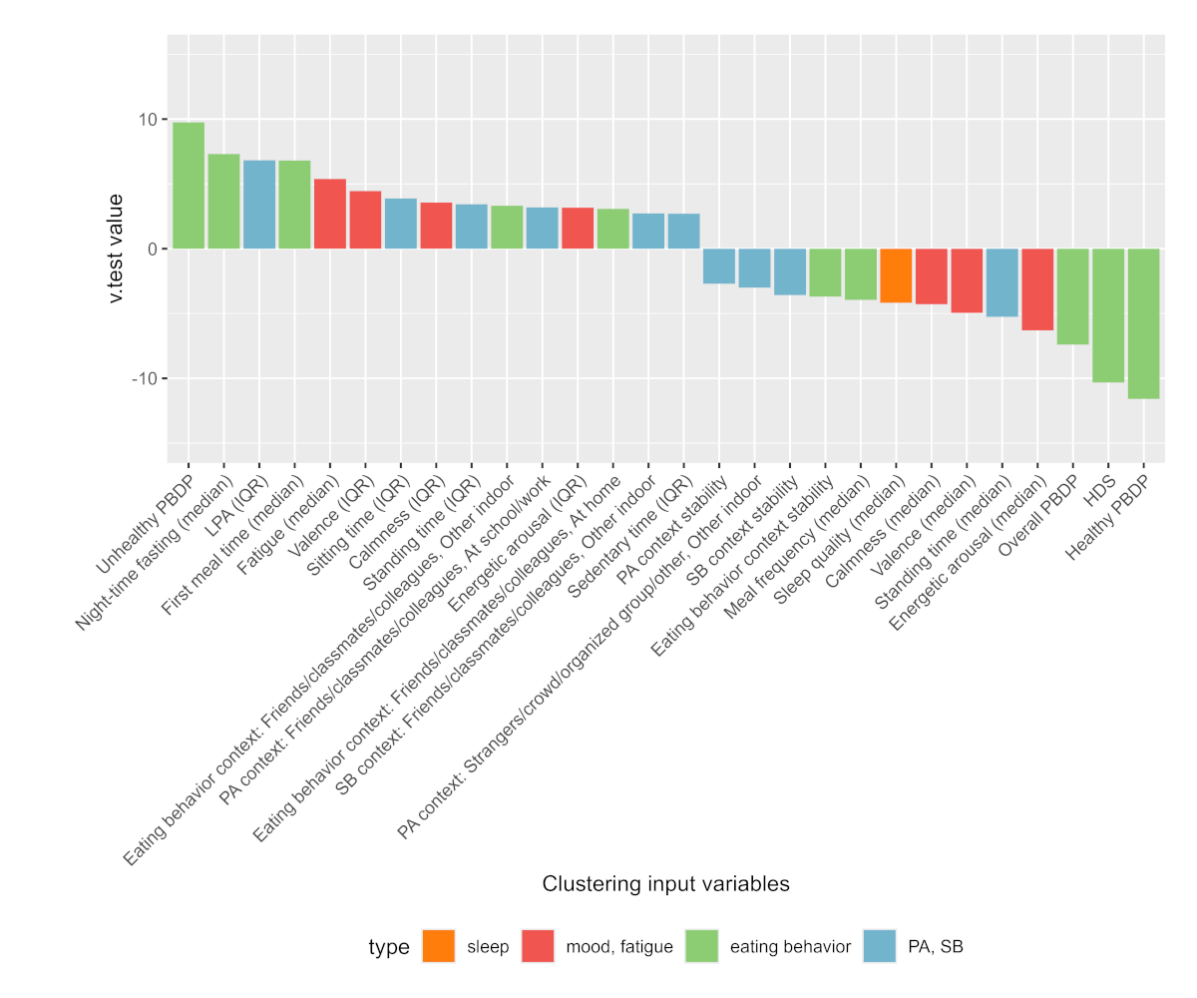
Characteristics of unhealthy behavior cluster; only variables with *P*<.05 were presented (n=109); the European Wearable Sensor Assessment of Physical and Eating Behaviors (WEALTH) study. Exact values for each bar are presented in Table S4 in Multimedia Appendix 1. HDS: healthy diet score; LPA: light physical activity; PA: physical activity; PBDP: plant-based diet propensity score; SB: sedentary behavior.

Based on FAMD results for inertia, MSEP (Figures S2 in Multimedia Appendix 1), and variable contributions (Figures S3 in Multimedia Appendix 1), 3 principal components (cumulative variance contribution 13.0%) were retained for HCPC. Mood scores, sleep quality, and standing time contributed most to the first component; SB, PA, and contextual variables to the second; and eating behavior, context, and LPA to the third (Figures S3 in Multimedia Appendix 1). HCPC identified 4 clusters according to inertia gain, participant representation (Figure S4 in Multimedia Appendix 1), and interpretability:

Cluster 1, labeled as “Unhealthy behavior*”* (n=109), group is characterized by frequent intake of unhealthy plant- and animal-based foods, less frequent intake of healthy plant-based foods, low HDS, long night-time fasting duration, late first meals, and infrequent meals. Participants also exhibited high variability in LPA and sitting time, low standing time, high fatigue, low mood with high variability, and poor sleep quality. Eating behavior variables were the primary determinants of this cluster. Contextually, participants often ate with friends, classmates, and/or colleagues at home or indoors, engaged in PA at college or work, and performed SB in other indoor spaces. They rarely practiced PA with strangers or in a group in other indoor spaces. Contextual stability scores across all behaviors were low (Figure 2; Table S4 in Multimedia Appendix 1).

**Figure 3.**
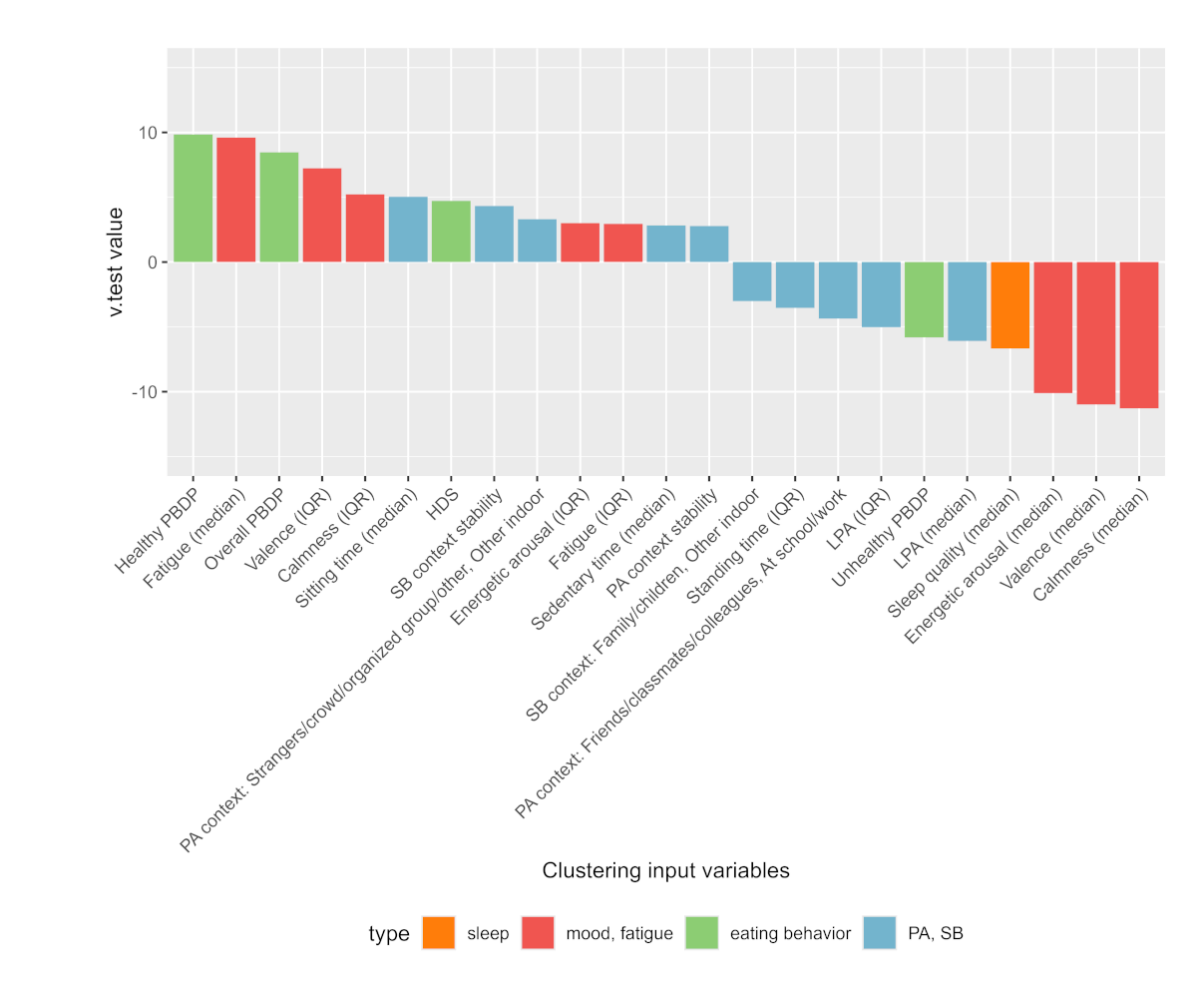
Characteristics of the mixed behavior–healthy diet cluster; only variables with *P*<.05 were presented (n=173); the European Wearable Sensor Assessment of Physical and Eating Behaviors (WEALTH) study. Exact values for each bar are presented in Table S4 in Multimedia Appendix 1. HDS: healthy diet score; LPA: light physical activity; PA: physical activity; PBDP: plant-based diet propensity score; SB: sedentary behavior.

Cluster 2, labeled as “Mixed behavior–healthy diet*”* (n=173), is characterized by low and variable mood, high, and variable fatigue, low sleep quality, high SB time, and low but stable LPA. HDS was high, with frequent intake of healthy plant-based foods and less frequent intake of unhealthy plant-based and animal-based foods. Mood and eating behavior variables were the primary determinants of this cluster. PA was often practiced with strangers or groups in indoor spaces, but less often with friends, classmates, and/or colleagues at college or work. SB and PA contexts were stable (Figure 3; Table S4 in Multimedia Appendix 1).

**Figure 4.**
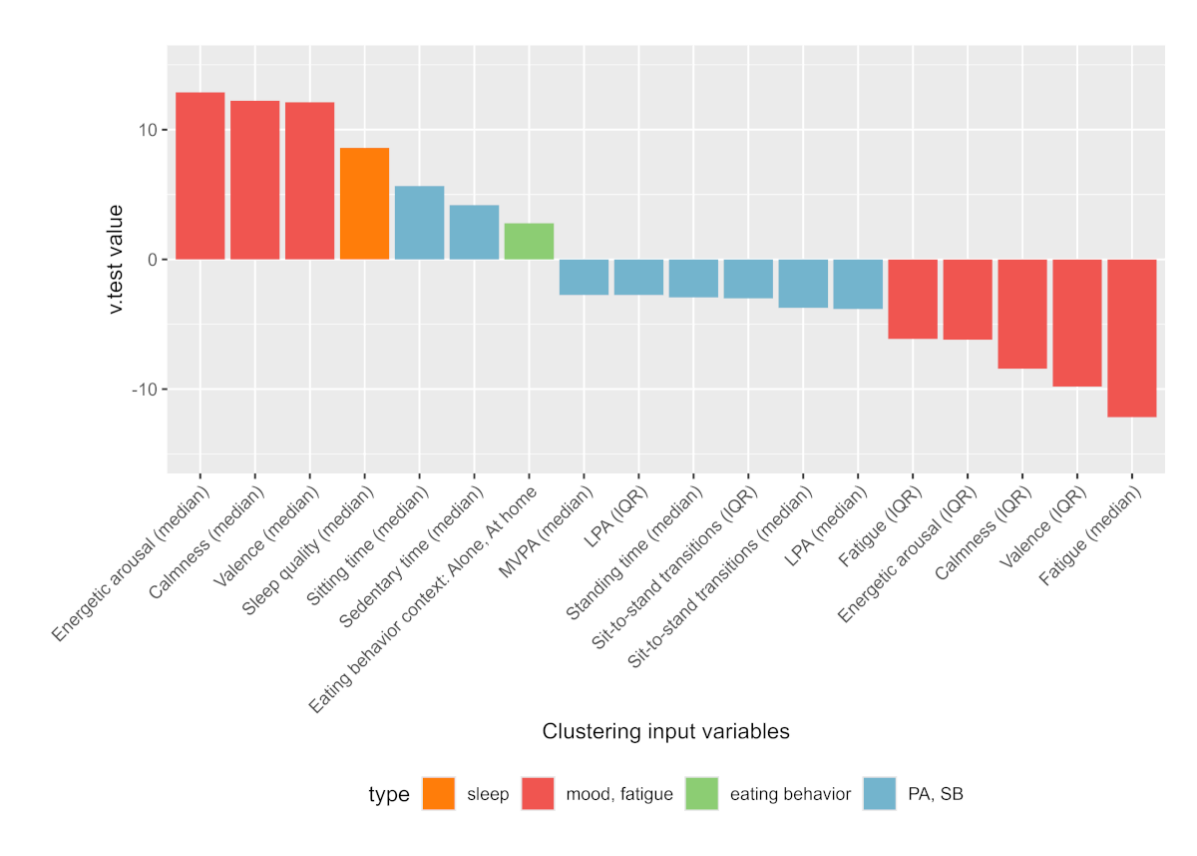
Characteristics of mixed behavior–stable good mood cluster; only variables with *P*<0.05 were presented (n=142); the European Wearable Sensor Assessment of Physical and Eating Behaviors (WEALTH) study. Exact values for each bar are presented in Table S4 in Multimedia Appendix 1. LPA: light physical activity; MVPA: moderate-to-vigorous physical activity; PA: physical activity; SB: sedentary behavior.

Cluster 3, labeled as “Mixed behavior–stable good mood*”* (n=142), is characterized by high and stable mood scores, low fatigue, high sleep quality, high SB, low and stable LPA, low MVPA, and low and stable sit-to-stand transitions. Mood variables were the primary determinants of this cluster. Participants frequently ate alone at home (Figure 4; Table S4 in Multimedia Appendix 1).

**Figure 5.**
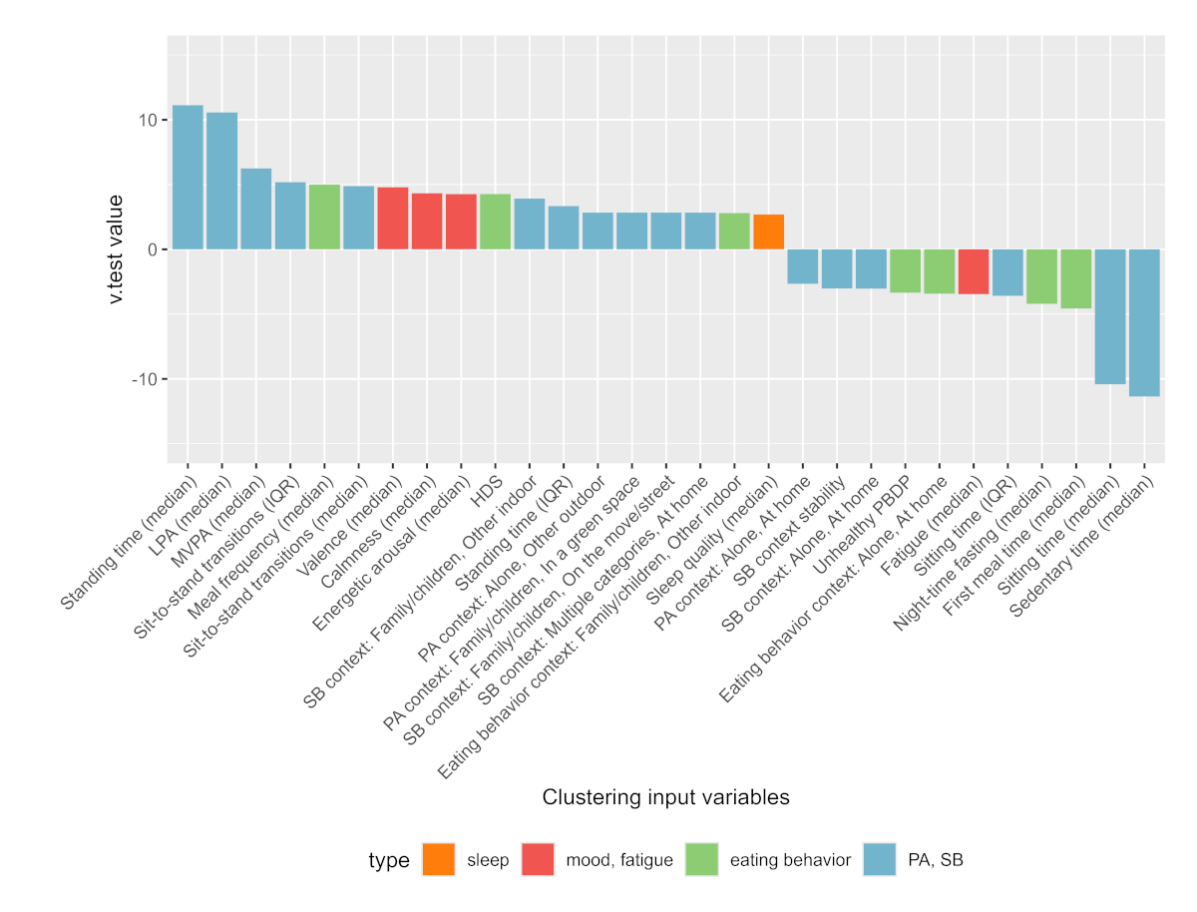
Characteristics of the healthy behavior cluster; only variables with *P*<.05 were presented (n=86); the European Wearable Sensor Assessment of Physical and Eating Behaviors (WEALTH) study. Exact values for each bar are presented in Table S4 in Multimedia Appendix 1. HDS: healthy diet score; LPA: light physical activity; MVPA: moderate to vigorous physical activity; PA: physical activity; PBDP: plant-based diet propensity score; SB: sedentary behavior.

Cluster 4, labeled as “Healthy behavior*”* (n=86), is characterized by high and variable standing time, high LPA and MVPA, high and variable sit-to-stand transitions, low and stable SB, short night-time fasting duration, early first meals, and frequent meal consumption. Mood, sleep score, and HDS were high, with limited intake frequency of unhealthy plant-based foods. PA and SB variables were the primary determinants of this cluster. Participants were frequently accompanied by family in indoor spaces or while in transit, and were less often alone at home during SB. PA was typically performed alone in outdoor spaces or with family in green spaces, and less often alone at home. Eating behavior contexts often involved the presence of family members in indoor spaces, whereas eating alone at home was rare (Figure 5; Table S4 in Multimedia Appendix 1). Cluster-specific characteristics based on input variables are presented in Table S5 in Multimedia Appendix 1. Models with different number of components or clusters resulted in at least 1 cluster with fewer than 10 participants (not reported), precluding meaningful association analyses with health outcomes. Regarding cluster stability, for the 4-cluster model, Jaccard values ranged from 0.43 to 0.65; for the 3-cluster model, from 0.41 to 0.60; and for the 5-cluster model, from 0.30 to 0.65. Sociodemographic and health status of each cluster are described in Table 1. The unhealthy behavior cluster mainly included young females and students with a lower educational level, with an overrepresentation of Irish participants. The mixed behavior–healthy diet cluster included mainly females with a median age of 35 (IQR 27.0-46.0) years and a high educational level, with an overrepresentation of German and French participants. The mixed behavior*–*stable good mood cluster included mainly older males with a high educational level, with an overrepresentation of French participants. The healthy behavior cluster included mainly older participants of both sexes with a medium educational level, with an overrepresentation of Czech participants.

### Association Analyses of Multibehavioral Clusters With Selected Health-Related Outcomes

**Figure 6.**
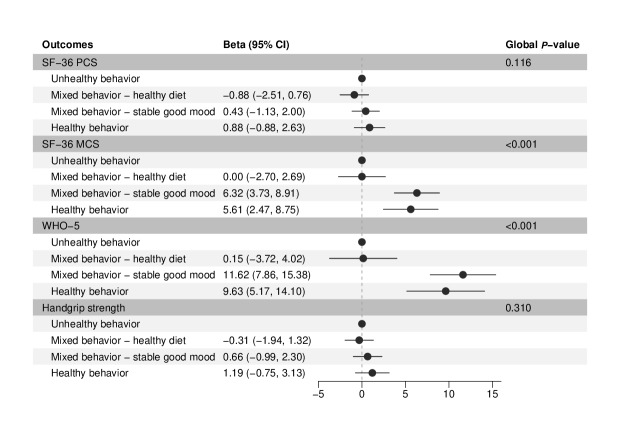
Associations between multibehavioral patterns and health-related continuous outcomes (N=510); the European Wearable Sensor Assessment of Physical and Eating Behaviours (WEALTH) project. MCS: mental composite score; PCS: physical composite score; SF-36: 36-Item Short Form Health Survey; WHO: World Health Organization; WHO-5: 5-item World Health Organization Well-Being Index.

**Figure 7.**
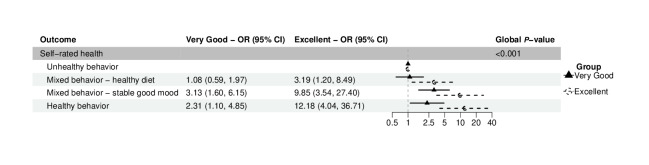
Association between multi-behavioral clusters and self-rated health (N=510); the European Wearable Sensor Assessment of Physical and Eating Behaviours (WEALTH) project. OR: odds ratio.

No substantial interactions were detected between multibehavioral clusters and age or sex (not reported); thus, all outcome analyses were conducted without stratification. Regression models adjusted for sociodemographic and anthropometric variables, smoking status, and alcohol consumption identified significant associations between multibehavioral clusters and SF-36 MCS, WHO-5 score, and self-rated health, but not with handgrip strength or SF-36 PCS (Figures 6 and 7). Compared with participants assigned to the unhealthy behavior cluster as the reference, those in the mixed behavior–stable good mood cluster had a mean SF-36 MCS of 6.32 (95% CI 3.73‐8.91) points higher, while participants in the healthy behavior cluster scored 5.61 (95% CI 2.47‐8.75) points higher. WHO-5 scores showed similar associations, with mean differences of +11.62 (95% CI 7.86‐15.38) in the mixed behavior*–*stable good mood cluster and +9.63 (95% CI 5.17‐14.10) in the healthy behavior cluster compared to the unhealthy behavior cluster. The mixed behavior*–*healthy diet cluster showed no significant difference in these 2 outcomes (Figure 6). Regarding self-rated health, the mixed behavior*–*stable good mood and healthy behavior clusters were more likely to report very good health (odds ratio [OR] 3.13, 95% CI 1.60‐6.15 and OR 2.31, 95% CI 1.10‐4.85, respectively) relative to poor, fair, or good health compared to those in the unhealthy behavior cluster. The likelihoods were even greater for reporting excellent health: OR 3.19 (95% CI 1.20‐8.49) for the mixed behavior*–*healthy diet cluster, OR 9.85 (95% CI 3.54‐27.40) for the mixed behavior*–*stable good mood cluster, and OR 12.18 (95% CI 4.04‐36.71) for the healthy behavior cluster (Figure 7).

## Discussion

### Principal Findings

This study identified 4 contrasting multibehavioral and contextual clusters in European adults by combining data from EMA, body-worn sensors, and the FFQ. The clusters differed in sociodemographic composition. In addition, compared with the cluster characterized by unhealthy behaviors, 2 clusters—one reflecting overall healthy behavior and another defined by stable positive mood despite mixed behavior—were positively associated with the mental component of HRQOL, self-rated health, and general well-being.

### Comparison With Prior Work

The distribution of participants was relatively balanced across clusters, with mixed behavior clusters being the most frequent and the unhealthy behavior cluster more prevalent than the healthy behavior cluster. This suggests the existence of relative combinations of both healthy and unhealthy behaviors, which coexist alongside predominantly healthy or unhealthy patterns. These findings are consistent with a systematic review of multibehavioral patterns (PA, SB, and eating behaviors) which identified three main categories: unhealthy (low MVPA, low diet quality, and high SB), mixed (combinations of healthy and unhealthy behaviors), and healthy (high MVPA, high diet quality, and low SB), with mixed patterns being the most frequent [5]. Another review focusing on PA, SB, sleep, and eating behavior also reported that unhealthy patterns (high SB, high ultraprocessed food, and low sleep) and mixed patterns (low PA, high SB, and satisfactory sleep) were the most common [6]. Overall, variables belonging to the same behavioral category varied in a consistent direction. For example, clusters characterized by longer PA duration exhibited shorter SB duration. Similarly, clusters associated with high diet quality were also marked by shorter night-time fasting and earlier first meals (healthy behavior cluster), and vice versa (unhealthy behavior cluster), aligning with data showing that such characteristics appear favorable to health [41].

A novel aspect of this clustering approach was the inclusion of mood and fatigue states captured through EMA. The three mood dimensions consistently varied in the same direction across clusters, such that all scores were either elevated or reduced simultaneously. Fatigue scores consistently demonstrated an inverse relationship with the 3 mood dimension scores across clusters. These variables were key contributors, particularly in the 2 mixed clusters, where mood and fatigue scores had the highest v-values. The mixed behavior–healthy diet cluster was characterized by negative and variable mood-fatigue states, while the mixed behavior*–*stable good mood cluster displayed positive and stable states, despite differences in PA, SB, eating behavior, and sleep. Across all 4 clusters, a more favorable mood-fatigue state was consistently associated with better sleep quality, which aligns with the well-known inverse relationships between inadequate sleep and psychological well-being [42]. For the unhealthy and healthy behavior clusters, behavioral characteristics (specifically eating behavior for the unhealthy behavior cluster, PA, and SB variables for the healthy behavior cluster) were the principal contributors. We therefore consider that both psychological states and eating and physical behaviors contributed meaningfully to cluster differentiation, although their relative importance varied across clusters, rather than mood driving the solution as a whole. Indeed, behavioral characteristics also followed expected profiles: the unhealthy behavior cluster exhibited unhealthy PA, SB, and eating behavior, along with poor and variable mood-fatigue states. The healthy behavior cluster showed the opposite profile. Previous EMA studies have reported bidirectional associations between positive affect and increased PA, though not with diet [13], while systematic reviews have found that negative mood was associated with noncompliance with nutritional recommendations [43,44]. These results are comparable to the unhealthy and healthy behavior clusters observed in this study. Importantly, mixed clusters characterized either by poor and variable mood combined with a healthy diet (mixed behavior–healthy diet cluster), or by stable good mood but unhealthy PA and SB (mixed behavior*–*good and stable mood cluster) were more prevalent, emphasizing the importance of mood-fatigue states in the construction of clusters.

Another innovative aspect of this study was the inclusion of social and physical contexts in the clustering analysis. Participants in the healthy behavior cluster were often accompanied by family members, spending time in indoor and outdoor spaces, including green spaces, and were less frequently alone at home, with an unstable SB context. Evidence from a systematic review of 29 EMA studies supports these observations, showing that social presence was generally linked to increased PA and reduced SB, although findings varied across studies [45]. The same review also found that being outdoors vs indoors consistently correlated with more PA and less SB [45]. Further, a study on contextual stability using EMA demonstrated that stable physical contexts promoted MVPA, whereas unstable social contexts were linked to lower SB [9]. Extending this to eating behavior, EMA data indicated that in males and females, respectively, eating with others and eating outside of the home were associated with a healthier diet [46]. These results are consistent with the contextual profile of the healthy behavior cluster. Therefore, our results integrate existing knowledge on contexts previously reported separately for PA, SB, and eating behaviors, and identify specific populations and contextual characteristics relevant to health behavior research. Findings from EMA-based studies on microlevel contexts—including our results—are consistent with those from research on macrolevel contexts, such as neighborhood green space availability and social interactions during meals, both positively associated with healthier behaviors and outcomes [11,12,47]. However, differences in sociodemographic profiles may partly explain contextual variations across clusters. For example, the unhealthy behavior cluster included a higher proportion of young females, potentially contributing to unstable contexts and a higher frequency of social interactions (eg, friends, classmates, and/or colleagues) across PA, SB, and eating behavior. The distribution of participants across countries differed between clusters and may partly reflect differences in participant characteristics between countries [17]. For instance, the unhealthy behavior cluster included relatively few French participants, who were drawn from the NutriNet-Santé cohort, whose participants have been reported to be more health-conscious than the general French population [48]. Irish participants were younger than those from other countries [17], and were overrepresented in the unhealthy behavior cluster. Early adulthood is widely recognized as a vulnerable period for the adoption of unhealthy lifestyle behaviors [49,50]. In addition, the recruitment incentive used in Czechia, Germany, and Ireland (€30–€40) may have contributed to differences in participant profiles compared with those enrolled in France [17]. Importantly, all countries were represented across all clusters, suggesting that the clustering structure captured broader multidimensional participant patterns rather than country membership alone.

Regarding health outcomes, significant positive associations were observed for self-rated health encompassing physical and mental health, general well-being, and mental HRQOL, notably for the mixed behavior*–*stable good mood and healthy behavior clusters, compared with the unhealthy behavior cluster. The mixed behavior*–*stable good mood cluster highlights the potential role of positive and stable mood-fatigue states (temporal dynamics) in relation to these outcomes, beyond PA, SB, and eating behavior. Previous research has suggested that consistent positive mood is strongly related to general well-being, while highly variable mood—despite occasional positive states—may relate to poorer health than stable positive mood [51]. Although the healthy behavior cluster also exhibited positive mood-fatigue states, this cluster was more strongly characterized by physical and eating behaviors, which suggests that good practices in physical and eating behaviors, along with positive and stable mood-fatigue states, are significantly associated with better health-related outcomes. Indeed, previous studies have reported that engaging in multiple poor lifestyle behaviors was associated with lower HRQOL or poor self-rated health, whereas a greater number of healthy behaviors or adherence to healthy behavioral patterns was associated with improved mental HRQOL, even though included behaviors varied across studies [52-55]. However, individuals with better health status may be more likely to engage in healthy physical and eating behaviors and to experience stable positive mood states, rather than the reverse. Only one significant association was noted, similar to these findings, for the mixed behavior*–*healthy diet cluster, showing a higher likelihood of reporting excellent self-rated health relative to poor, fair, or good compared to the unhealthy behavior cluster. This suggests that while healthy eating behavior alone may not fully compensate for other unfavorable behaviors in terms of mental health, it may positively influence perceived physical health. However, no significant association was observed for the physical health outcomes (handgrip strength, physical HRQOL) selected in this study. Given prior findings linking multibehavioral patterns with physical health [5], further longitudinal research with other outcome measures may help clarify the significance of the result of higher likelihood of reporting excellent self-rated health in the mixed behavior–healthy diet cluster.

Overall, our results can be viewed as a proof of concept for integrating EMA and body-worn movement sensor data to identify multibehavioral clusters, demonstrating its feasibility in 4 European countries and producing results that align with current knowledge on lifestyle behaviors and their relationships with health-related outcomes.

### Limitations and Strengths

Several limitations must be acknowledged. First, the use of a convenience sample as recruited here in 4 European countries limits generalizability. Participants in the WEALTH study may have greater interest in physical and eating behaviors in relation to health than the general population, potentially reducing variability and skewing patterns in key behaviors and health outcomes. Thus, observed associations could be more pronounced in the general population. Second, in order to include repeated and nonrepeated measures in clustering, aggregated indicators (ie, median and IQR) were used for repeated variables, resulting in the loss of temporal sequencing and dynamic components (eg, how they change over time, seasonality, cyclicity of the behaviors). Third, despite adjustments for confounding variables, residual confounding may remain. Measurement errors may also have contributed to underestimating associations. Fourth, the 3 principal components included in HCPC explained only 13.0% of the total variability. In this study, 35 variables were included in FAMD, among which social and physical contexts alone comprised 30 modalities for each behavioral domain (PA, SB, and eating behavior). In such settings, each individual component captures a small proportion of total variance by design, and cumulative explained variance across a limited number of components is inherently modest. However, it should be noted that the 3 principal components covered all input categories (ie, sleep, PA, SB, eating behavior, and contextual characteristics), which supports their representativeness of the multidimensional behavioral space. In addition, the number of components was selected based on the Scree plot of eigenvalues and the MSEP obtained through k-fold cross-validation. Regarding cluster stability, we acknowledge that several clusters across all models fall below the conventional stability threshold of 0.60, indicating mixed stability. We nonetheless retained the 4-cluster model because it yielded the highest minimum Jaccard values across competing models, which was supported by the gain-in-inertia plot, and produced clusters with sufficient sample size for subsequent regression analyses. Therefore, the identified clusters should be interpreted as exploratory profiles that warrant confirmation in future studies rather than as definitive phenotypes. Finally, the cross-sectional study design prevents identifying whether behavioral and contextual patterns influence health-related outcomes or vice-versa. Longitudinal studies in representative population samples are necessary to clarify the directionality of the observed associations. Despite these limitations, this study has important strengths. To our knowledge, this is the first study in a relatively large sample of adults from 4 European countries to examine multibehavioral clusters, including PA, SB, sleep, eating behavior, and their contexts in relation to health-related outcomes. Psychological, social, and physical contexts were repeatedly assessed, while PA and SB were continuously monitored over 7 days using EMA and wearable devices, respectively, allowing for the assessment of within-person variability. In addition, the use of the HealthReact platform allowed seamless coupling of EMA with wearable sensor data, supporting real-time and context-sensitive assessment of daily behaviors.

### Conclusions

In this study, 4 distinct multibehavioral clusters were identified, each shaped by psychological, social, and physical contextual characteristics, and significantly associated with mental HRQOL, overall well-being, and self-rated health. These findings support the feasibility of identifying important multibehavioral patterns for health by using EMA and body-worn movement sensors. The results indicate that engaging in healthy behaviors across multiple lifestyle domains is associated with more favorable health outcomes than engaging in healthy practices within a single domain. Understanding these associations is critical for the precise identification of high-risk population subgroups and for informing the development of targeted interventions tailored to specific populations and contextual environments [56]. A promising direction for future research involves further investigating sequential patterns across behaviors and their contexts in longitudinal study designs, which may provide deeper insights into multibehavioral dynamics and help develop targeted prevention strategies.

## Supplementary material

10.2196/85991 Multimedia Appendix 1 Additional details on food categorization for plant-based propensity score, comparison of participant characteristics and clustering input variables between included and excluded participants, cluster characteristics, participant flow, and factor analysis and clustering results.

## Abbreviations

EMA: ecological momentary assessment
FAMD: factor analysis for mixed data
FFQ: Food Frequency Questionnaire
HCPC: hierarchical clustering on principal components
HDS: healthy diet score
HRQOL: health-related quality of life
ISCED: International Standard Classification of Education
MCS: mental composite score
MSEP: mean squared error of prediction
MVPA: moderate-to-vigorous physical activity
OR: odds ratio
PA: physical activity
PBDP: plant-based diet propensity
PCS: physical composite score
SB: sedentary behavior
SF-36: 36-Item Short Form Health Survey
WEALTH: Wearable Sensor Assessment of Physical and Eating Behaviours
WHO-5: 5-item World Health Organization Well-Being Index
