# Supplementary material for: Behavioral and Contextual Patterns Derived From Ecological Momentary Assessment and Wearable Sensors and Their Associations With Health-Related Outcomes: WEALTH Cross-Sectional Study
Source: JMIR Form Res. 2026 Aug 11;10:e85991. doi: 10.2196/85991 (PMC13460797; doi:10.2196/85991)
Supplement: Supplementary file 1 [file formative-v10-e85991-s001.docx]

**Supplementary Table S1:** Categorization of foods from 62-item Food Frequency Questionnaire for Plant-Based Propensity Score

| **Healthy plant-based foods** | | **Unhealthy plant-based foods** | | **Animal-based foods** | |
| --- | --- | --- | --- | --- | --- |
| Whole grains | Porridge; oatmeal; gruel; unsweetened cereals and muesli; whole meal bread; dark roll; dark crispbread; whole meal pasta; noodles; brown rice; other unrefined cereals | Refined grains | Breakfast cereals; sweetened muesli and cereals; white bread, roll, crispbread, pasta, noodles, and rice; dish or milled cereals | Animal fat | Butter or margarine, reduced-fat products on bread |
| Fruits | Fruits without added sugars | Snacks | Fried potatoes; potato croquettes; ketchup; crisps; corn crisps; popcorn | Dairy | Plain unsweetened milk; sweetened milk; milky desserts; plain unsweetened yoghurt or kefir; sweet yoghurt; fermented milk; sliced cheese; spreadable cheese; crated cheese; fat-reduced cheese |
| Vegetables | Cooked vegetables; boiled potatoes; beans; legumes; raw vegetables; meat replacement products (tofu, tempe, quorn) | Sugar-sweetened beverages | Fruit juices; sugar-sweetened drinks not carbonated; carbonated sugar-sweetened drinks; diet drinks, artificially sweetened drinks not carbonated; sweetened coffee and tea | Egg | Fried, scrambled, boiled or poached eggs |
| Nuts | Nuts; seeds; dried fruits | Sweets and desserts | Fruits with added sugars; jam; honey; chocolate or nut-based spread; chocolate; candy bars; milk or fruit-based bars; candies; loose candies; marshmallows; biscuits; packaged cakes; pastries; puddings | Fish or seafood | Boiled, grilled, oven-baked or raw not fried fish; fried and/or coated fish; canned fish |
| Vegetable oils | Olive oil |  |  | Meat | Cold cuts and preserved, ready to cook meat product; not fried fresh meat; fried meat; fresh meat; not fried cooked poultry; fried poultry |
| Tea and coffee | Unsweetened coffee and tea |  |  | Miscellaneous | Mayonnaise; Mayonnaise-based products; pizza as main dish; hamburger; hot dog; kebab; wrap; falafel; savory pastries; fritters; ice cream |
| Plant-based milk and yoghurt | Yoghurt and drink based on vegan milk; substitutes or plant milks and yoghurt (e.g. soy, almond, rice, oat or coconut drinks and yoghurts) |  |  |  |  |

**Supplementary Table S2**: Comparison of sociodemographic and health characteristics between included (n= 510) and excluded (n= 117) participants; the WEALTH study

| **Characteristics** | **Missing** (N) | **Overall**^1^ N = 627 | **Excluded**^1^  N = 117 | **Included**^1^  N = 510 | **P-value** |
| --- | --- | --- | --- | --- | --- |
| **Sex** | 0 |  |  |  | 0.579^2^ |
| Female |  | 56.1 (352) | 53.8 (63) | 56.7 (289) |  |
| Male |  | 43.9 (275) | 46.2 (54) | 43.3 (221) |  |
| **Country** | 0 |  |  |  | 0.234^2^ |
| Czechia |  | 23.9 (150) | 27.4 (32) | 23.1 (118) |  |
| France |  | 24.9 (156) | 17.9 (21) | 26.5 (135) |  |
| Germany |  | 25.8 (162) | 25.6 (30) | 25.9 (132) |  |
| Ireland |  | 25.4 (159) | 29.1 (34) | 24.5 (125) |  |
| **Age** | 0 | 36.0 (25.0, 50.0) | 37.0 (26.0, 49.0) | 35.5 (25.0, 50.0) | 0.535^3^ |
| **Educational Level** | 16 |  |  |  | 0.067^2^ |
| < High School Degree |  | 21.1 (129) | 21.8 (22) | 21.0 (107) |  |
| High School Degree |  | 27.0 (165) | 28.7 (29) | 26.7 (136) |  |
| > High School Degree |  | 46.3 (283) | 49.5 (50) | 45.7 (233) |  |
| Others |  | 5.6 (34) | 0.0 (0) | 6.7 (34) |  |
| **Occupation** | 14 |  |  |  | 0.561^4^ |
| Student |  | 30.8 (189) | 32.0 (33) | 30.6 (156) |  |
| Employed |  | 61.5 (377) | 58.3 (60) | 62.2 (317) |  |
| Non-employed |  | 3.3 (20) | 2.9 (3) | 3.3 (17) |  |
| Others |  | 4.4 (27) | 6.8 (7) | 3.9 (20) |  |
| **Body Mass Index** | 0 | 23.9 (21.6, 26.6) | 23.9 (21.8, 26.5) | 24.0 (21.6, 26.6) | 0.479^3^ |
| **Smoking status** | 14 |  |  |  | 0.595^2^ |
| Current smoker |  | 7.3 (45) | 9.7 (10) | 6.9 (35) |  |
| Ex-smoker |  | 17.5 (107) | 17.5 (18) | 17.5 (89) |  |
| Nonsmoker |  | 75.2 (461) | 72.8 (75) | 75.7 (386) |  |
| **Alcohol consumption** | 17 |  |  |  | 0.067^2^ |
| >= 4 times per week |  | 7.5 (46) | 2.0 (2) | 8.6 (44) |  |
| 1-3 times per week |  | 39.0 (238) | 43.0 (43) | 38.2 (195) |  |
| 0 times per week |  | 53.4 (326) | 55.0 (55) | 53.1 (271) |  |
| **SF-36 PCS** | 14 | 55.9 (52.6, 58.6) | 55.1 (51.9, 59.0) | 56.0 (52.9, 58.6) | 0.567^3^ |
| **SF-36 MCS** | 14 | 49.9 (41.3, 54.5) | 49.1 (41.0, 55.0) | 50.1 (41.4, 54.5) | 0.658^3^ |
| **WHO-5 Score** | 13 | 64.0 (52.0, 76.0) | 64.0 (52.0, 76.0) | 64.0 (52.0, 76.0) | 0.874^3^ |
| **Handgrip strength, Median (Q1, Q3)** | 0 | 32.7 (26.9, 43.2) | 33.2 (27.7, 45.0) | 32.5 (26.9, 43.0) | 0.286^3^ |
| **Self-rated health** | 13 |  |  |  | 0.753^2^ |
| Poor / fair / good |  | 35.7 (219) | 33.7 (35) | 36.1 (184) |  |
| Very good |  | 46.6 (286) | 46.2 (48) | 46.7 (238) |  |
| Excellent |  | 17.8 (109) | 20.2 (21) | 17.3 (88) |  |
| ^1^% (n) for categorical variables; Median (Q1, Q3) for continuous variables | | | | | |
| ^2^Pearson's Chi-squared test: Included vs Excluded | | | | | |
| ^3^Wilcoxon rank sum test: Included vs Excluded | | | | | |
| ^4^Fisher's exact test: Included vs Excluded | | | | | |
| MCS: mental composite score, PCS: physical composite score, SF-36: short form 36 health survey questionnaire, WHO: world health organization | | | | | |

**Supplementary Table S3**: Comparison of clustering input variables between Included (n= 510) and excluded (n= 117) participants; the WEALTH study

| **Variable** | **Mis- sing** (N) | **Overall**^1^ N = 627 | **Excluded**^1^  N = 117 | **Included**^1^  N = 510 | **P-value** |
| --- | --- | --- | --- | --- | --- |
| **Sleep quality median** | 22 | 66.0 (54.0, 79.0) | 67.0 (53.5, 80.0) | 66.0 (54.0, 79.0) | 0.572^2^ |
| **Sleep duration median** | 23 | 7.0 (6.5, 7.6) | 7.1 (6.5, 7.8) | 7.0 (6.5, 7.6) | 0.574^2^ |
| **Fatigue** |  |  |  |  |  |
| Median | 2 | 34.0 (22.0, 46.5) | 34.0 (22.0, 46.5) | 34.0 (22.0, 47.0) | 0.731^2^ |
| IQR | 2 | 19.0 (12.5, 26.5) | 16.5 (12.3, 25.5) | 19.3 (12.5, 27.0) | 0.164^2^ |
| **Valence** |  |  |  |  |  |
| Median | 2 | 76.0 (66.0, 85.5) | 75.0 (65.5, 84.5) | 76.4 (66.5, 86.0) | 0.072^2^ |
| IQR | 2 | 11.0 (7.0, 17.0) | 10.9 (6.5, 17.0) | 11.0 (7.0, 17.0) | 0.979^2^ |
| **Calmness** |  |  |  |  |  |
| Median | 2 | 73.5 (63.5, 83.0) | 71.0 (62.3, 82.3) | 73.5 (63.5, 83.0) | 0.183^2^ |
| IQR | 2 | 12.1 (7.8, 19.5) | 13.0 (7.8, 19.5) | 12.0 (7.6, 19.5) | 0.498^2^ |
| **Energetic arousal** |  |  |  |  |  |
| Median | 2 | 65.5 (53.8, 78.0) | 63.5 (52.0, 74.5) | 66.0 (54.0, 78.8) | 0.138^2^ |
| IQR | 2 | 18.5 (12.0, 26.0) | 17.9 (11.0, 24.9) | 18.5 (12.5, 26.0) | 0.370^2^ |
| **PBDP (overall)** | 18 | 68.6 (61.1, 77.1) | 68.1 (61.7, 75.9) | 68.9 (61.0, 77.2) | 0.511^2^ |
| **PBDP (healthy)** | 18 | 50.6 (38.1, 62.7) | 50.0 (37.0, 61.3) | 50.7 (38.1, 63.3) | 0.625^2^ |
| **PBDP (unhealthy)** | 18 | 17.4 (11.0, 24.8) | 17.4 (10.8, 24.0) | 17.3 (11.1, 25.3) | 0.605^2^ |
| **Healthy Dietary Score** | 18 | 30.0 (23.0, 36.0) | 31.0 (23.0, 38.0) | 29.0 (23.0, 36.0) | 0.156^2^ |
| **Number of meals median** | 8 | 4.0 (3.0, 6.0) | 4.0 (3.0, 5.0) | 4.0 (3.0, 6.0) | 0.004^2^ |
| **First meal timing median** | 16 | 8.6 (7.7, 9.8) | 9.0 (8.0, 10.1) | 8.5 (7.7, 9.8) | 0.096^2^ |
| **Night-time fasting median** | 22 | 13.2 (11.9, 15.4) | 13.5 (12.0, 16.5) | 13.2 (11.8, 15.1) | 0.158^2^ |
| **LPA** |  |  |  |  |  |
| Median | 1 | 132.1 (106.4, 165.5) | 144.0 (114.8, 185.1) | 130.3 (104.3, 161.8) | 0.003^2^ |
| IQR | 1 | 42.8 (28.8, 62.3) | 47.7 (32.1, 67.9) | 42.1 (28.0, 61.8) | 0.106^2^ |
| **MVPA** |  |  |  |  |  |
| Median | 1 | 81.2 (62.6, 102.3) | 85.3 (61.5, 111.9) | 80.6 (62.7, 100.0) | 0.218^2^ |
| IQR | 1 | 35.5 (22.3, 47.8) | 34.7 (22.2, 44.6) | 35.5 (22.3, 48.3) | 0.354^2^ |
| **Sedentary time** |  |  |  |  |  |
| Median | 35 | 553.8 (486.2, 617.7) | 550.8 (475.7, 617.1) | 554.0 (489.3, 619.0) | 0.559^2^ |
| IQR | 35 | 118.3 (79.7, 163.0) | 119.1 (72.4, 165.2) | 118.3 (79.8, 162.8) | 0.858^2^ |
| **Standing time** |  |  |  |  |  |
| Median | 35 | 228.5 (182.3, 291.1) | 231.6 (181.4, 302.6) | 228.0 (182.6, 286.6) | 0.452^2^ |
| IQR | 35 | 71.0 (47.1, 100.3) | 74.6 (48.7, 102.0) | 70.6 (46.0, 99.9) | 0.599^2^ |
| **Sitting time** |  |  |  |  |  |
| Median | 35 | 467.5 (393.4, 538.7) | 456.8 (351.8, 530.1) | 468.3 (398.7, 539.3) | 0.174^2^ |
| IQR | 35 | 126.2 (88.6, 174.5) | 124.1 (87.9, 164.9) | 126.2 (89.0, 176.5) | 0.671^2^ |
| **Number of sit to stand transitions** |  |  |  |  |  |
| Median | 35 | 50.8 (41.5, 60.0) | 53.8 (44.0, 66.0) | 50.0 (41.0, 60.0) | 0.005^2^ |
| IQR | 35 | 11.0 (8.0, 16.5) | 12.0 (9.0, 19.8) | 11.0 (8.0, 16.0) | 0.021^2^ |
| **Eating behavior context stability** | 11 | -1.9 (-2.3, -1.6) | -2.1 (-2.5, -1.6) | -1.9 (-2.2, -1.5) | 0.020^2^ |
| **PA context stability** | 27 | -2.9 (-3.2, -2.4) | -2.9 (-3.3, -2.4) | -2.9 (-3.2, -2.4) | 0.602^2^ |
| **SB context stability** | 34 | -2.0 (-2.6, -1.5) | -2.3 (-3.1, -1.6) | -2.0 (-2.5, -1.5) | 0.008^2^ |
| **Most frequent eating behavior context modality** | 8 |  |  |  | 0.114^3^ |
| Alone, At home |  | 51.4 (318) | 55.0 (60) | 50.6 (258) |  |
| Alone, At college or work |  | 3.9 (24) | 1.8 (2) | 4.3 (22) |  |
| Alone, In a green space |  | 0.2 (1) | 0.9 (1) | 0.0 (0) |  |
| Alone, Other indoor |  | 1.0 (6) | 1.8 (2) | 0.8 (4) |  |
| Family/children, At home |  | 26.0 (161) | 22.9 (25) | 26.7 (136) |  |
| Family/children, In a green space |  | 0.2 (1) | 0.0 (0) | 0.2 (1) |  |
| Family/children, On the move / on the street |  | 0.2 (1) | 0.9 (1) | 0.0 (0) |  |
| Family/children, Other indoor |  | 4.4 (27) | 1.8 (2) | 4.9 (25) |  |
| Friends/classmates/colleagues, At home |  | 1.3 (8) | 1.8 (2) | 1.2 (6) |  |
| Friends/classmates/colleagues, At college or work |  | 4.0 (25) | 4.6 (5) | 3.9 (20) |  |
| Friends/classmates/colleagues, In a green space |  | 0.2 (1) | 0.0 (0) | 0.2 (1) |  |
| Friends/classmates/colleagues, On the move / on the street |  | 0.3 (2) | 0.0 (0) | 0.4 (2) |  |
| Friends/classmates/colleagues, Other indoor |  | 2.9 (18) | 5.5 (6) | 2.4 (12) |  |
| Multiple categories, At home |  | 3.6 (22) | 1.8 (2) | 3.9 (20) |  |
| Multiple categories, In a green space |  | 0.2 (1) | 0.0 (0) | 0.2 (1) |  |
| Multiple categories, Other indoor |  | 0.2 (1) | 0.9 (1) | 0.0 (0) |  |
| Strangers/crowd/organized group/other, At college or work |  | 0.2 (1) | 0.0 (0) | 0.2 (1) |  |
| Strangers/crowd/organized group/other, Other indoor |  | 0.2 (1) | 0.0 (0) | 0.2 (1) |  |
| **Most frequent PA context modality** | 15 |  |  |  | 0.929^3^ |
| Alone, At home |  | 16.5 (101) | 18.6 (19) | 16.1 (82) |  |
| Alone, At college or work |  | 9.5 (58) | 9.8 (10) | 9.4 (48) |  |
| Alone, In a green space |  | 3.8 (23) | 4.9 (5) | 3.5 (18) |  |
| Alone, On the move / on the street |  | 22.7 (139) | 22.5 (23) | 22.7 (116) |  |
| Alone, Other indoor |  | 6.7 (41) | 3.9 (4) | 7.3 (37) |  |
| Alone, Other outdoor |  | 2.8 (17) | 2.9 (3) | 2.7 (14) |  |
| Family/children, At home |  | 5.9 (36) | 6.9 (7) | 5.7 (29) |  |
| Family/children, In a green space |  | 0.7 (4) | 1.0 (1) | 0.6 (3) |  |
| Family/children, On the move / on the street |  | 2.8 (17) | 2.9 (3) | 2.7 (14) |  |
| Family/children, Other indoor |  | 4.2 (26) | 2.0 (2) | 4.7 (24) |  |
| Family/children, Other outdoor |  | 0.3 (2) | 1.0 (1) | 0.2 (1) |  |
| Friends/classmates/colleagues, At home |  | 0.3 (2) | 0.0 (0) | 0.4 (2) |  |
| Friends/classmates/colleagues, At college or work |  | 6.5 (40) | 6.9 (7) | 6.5 (33) |  |
| Friends/classmates/colleagues, In a green space |  | 0.5 (3) | 0.0 (0) | 0.6 (3) |  |
| Friends/classmates/colleagues, On the move / on the street |  | 1.5 (9) | 0.0 (0) | 1.8 (9) |  |
| Friends/classmates/colleagues, Other indoor |  | 3.6 (22) | 3.9 (4) | 3.5 (18) |  |
| Friends/classmates/colleagues, Other outdoor |  | 1.1 (7) | 2.0 (2) | 1.0 (5) |  |
| Multiple categories, At home |  | 0.3 (2) | 0.0 (0) | 0.4 (2) |  |
| Multiple categories, At college or work |  | 0.2 (1) | 0.0 (0) | 0.2 (1) |  |
| Multiple categories, In a green space |  | 0.2 (1) | 0.0 (0) | 0.2 (1) |  |
| Multiple categories, On the move / on the street |  | 0.3 (2) | 0.0 (0) | 0.4 (2) |  |
| Multiple categories, Other indoor |  | 0.7 (4) | 2.0 (2) | 0.4 (2) |  |
| Multiple categories, Other outdoor |  | 0.7 (4) | 0.0 (0) | 0.8 (4) |  |
| Strangers/crowd/organized group/other, At home |  | 0.2 (1) | 0.0 (0) | 0.2 (1) |  |
| Strangers/crowd/organized group/other, In a green space |  | 0.2 (1) | 0.0 (0) | 0.2 (1) |  |
| Strangers/crowd/organized group/other, On the move / on the street |  | 2.8 (17) | 2.0 (2) | 2.9 (15) |  |
| Strangers/crowd/organized group/other, Other indoor |  | 5.1 (31) | 6.9 (7) | 4.7 (24) |  |
| Strangers/crowd/organized group/other, Other outdoor |  | 0.2 (1) | 0.0 (0) | 0.2 (1) |  |
| **Most frequent SB context modality** | 16 |  |  |  | 0.038^3^ |
| Alone, At home |  | 54.8 (335) | 56.4 (57) | 54.5 (278) |  |
| Alone, At college or work |  | 11.1 (68) | 6.9 (7) | 12.0 (61) |  |
| Alone, In a green space |  | 0.5 (3) | 3.0 (3) | 0.0 (0) |  |
| Alone, On the move / on the street |  | 0.7 (4) | 1.0 (1) | 0.6 (3) |  |
| Alone, Other indoor |  | 1.8 (11) | 2.0 (2) | 1.8 (9) |  |
| Family/children, At home |  | 10.8 (66) | 6.9 (7) | 11.6 (59) |  |
| Family/children, At college or work |  | 0.2 (1) | 1.0 (1) | 0.0 (0) |  |
| Family/children, In a green space |  | 0.2 (1) | 0.0 (0) | 0.2 (1) |  |
| Family/children, On the move / on the street |  | 0.7 (4) | 1.0 (1) | 0.6 (3) |  |
| Family/children, Other indoor |  | 3.8 (23) | 3.0 (3) | 3.9 (20) |  |
| Friends/classmates/colleagues, At home |  | 0.5 (3) | 0.0 (0) | 0.6 (3) |  |
| Friends/classmates/colleagues, At college or work |  | 11.1 (68) | 13.9 (14) | 10.6 (54) |  |
| Friends/classmates/colleagues, In a green space |  | 0.2 (1) | 0.0 (0) | 0.2 (1) |  |
| Friends/classmates/colleagues, Other indoor |  | 1.6 (10) | 3.0 (3) | 1.4 (7) |  |
| Multiple categories, At home |  | 0.7 (4) | 1.0 (1) | 0.6 (3) |  |
| Multiple categories, At college or work |  | 0.2 (1) | 0.0 (0) | 0.2 (1) |  |
| Multiple categories, Other indoor |  | 0.2 (1) | 1.0 (1) | 0.0 (0) |  |
| Strangers/crowd/organized group/other, At home |  | 0.2 (1) | 0.0 (0) | 0.2 (1) |  |
| Strangers/crowd/organized group/other, At college or work |  | 0.2 (1) | 0.0 (0) | 0.2 (1) |  |
| Strangers/crowd/organized group/other, Other indoor |  | 0.8 (5) | 0.0 (0) | 1.0 (5) |  |
| ^1^% (n) for categorical variables; Median (Q1, Q3) for continuous variables  ^2^Kruskal-Wallis rank sum test: Included vs Excluded  ^3^Fisher's Exact Test using Monte-Carlo approach with simulated p-value (1e+05 replicates) across multi-behavioral patterns: Included vs Excluded  IQR: interquartile range, HDS: healthy diet score, LPA: light physical activity, MVPA: Moderate to vigorous physical activity, PA: physical activity, PBDP: plant-based diet propensity score, SB: sedentary behavior | | | | | |

**Supplementary Table S4**: Cluster characteristics showing clustering input variables with v values (N= 510); the WEALTH study

| **Variable** | ***Unhealthy behavior*** | ***Mixed behavior - healthy diet*** | ***Mixed behavior -***  ***stable good mood*** | ***Healthy behavior*** |
| --- | --- | --- | --- | --- |
| **Sleep quality (median)** | -4.18*** | -6.66*** | 8.60*** | 2.69** |
| **Fatigue** |  |  |  |  |
| Median | 5.39*** | 9.59*** | -12.18*** | -3.45*** |
| IQR |  | 2.95** | -6.13*** |  |
| **Valence** |  |  |  |  |
| Median | -4.95*** | -10.98*** | 12.12*** | 4.79*** |
| IQR | 4.45*** | 7.23*** | -9.80*** |  |
| **Calmness** |  |  |  |  |
| Median | -4.28*** | -11.29*** | 12.23*** | 4.32*** |
| IQR | 3.56*** | 5.21*** | -8.43*** |  |
| **Energetic arousal** |  |  |  |  |
| Median | -6.30*** | -10.11*** | 12.88*** | 4.26*** |
| IQR | 3.17** | 3.00** | -6.20*** |  |
| **Overall PBDP** | -7.39*** | 8.46*** |  |  |
| **Healthy PBDP** | -11.59*** | 9.84*** |  |  |
| **Unhealthy PBDP** | 9.74*** | -5.81*** |  | -3.36*** |
| **HDS** | -10.31*** | 4.72*** |  | 4.26*** |
| **Meal frequency (median)** | -3.95*** |  |  | 5.00*** |
| **First meal time (median)** | 6.80*** |  |  | -4.56*** |
| **Night-time fasting (median)** | 7.29*** |  |  | -4.20*** |
| **LPA** |  |  |  |  |
| Median |  | -6.09*** | -3.83*** | 10.56*** |
| IQR | 6.82*** | -5.02*** | -2.74** |  |
| **MVPA (median)** |  |  | -2.74** | 6.24*** |
| **Sedentary time** |  |  |  |  |
| Median |  | 2.82** | 4.19*** | -11.35*** |
| IQR | 2.70** |  |  |  |
| **Standing time** |  |  |  |  |
| Median | -5.25*** |  | -2.94** | 11.13*** |
| IQR | 3.43*** | -3.53*** |  | 3.34*** |
| **Sitting time** |  |  |  |  |
| Median |  | 5.03*** | 5.66*** | -10.41*** |
| IQR | 3.87*** |  |  | -3.59*** |
| **Number of sit-to-stand transitions** |  |  |  |  |
| Median |  |  | -3.74*** | 4.89*** |
| IQR |  |  | -3.00** | 5.19*** |
| **Eating behavior context stability** | -3.70*** |  |  |  |
| **PA context stability** | -2.70** | 2.79** |  |  |
| **SB context stability** | -3.58*** | 4.33*** |  | -3.02** |
| **Context Eating behavior** |  |  |  |  |
| Alone, At home |  |  | 2.79** | -3.43*** |
| Family/children, Other indoor |  |  |  | 2.80** |
| Friends/classmates/colleagues, At home | 3.07** |  |  |  |
| Friends/classmates/colleagues, Other indoor | 3.33*** |  |  |  |
| **Context PA** |  |  |  |  |
| Alone, At home |  |  |  | -2.66** |
| Alone, Other outdoor |  |  |  | 2.84** |
| Family/children, In a green space |  |  |  | 2.83** |
| Friends/classmates/colleagues, At college/work | 3.18** | -4.35*** |  |  |
| Strangers/crowd/organized group/other, Other indoor | -3.00** | 3.29*** |  |  |
| **Context SB** |  |  |  |  |
| Alone, At home |  |  |  | -3.03** |
| Family/children, On the move/street |  |  |  | 2.83** |
| Family/children, Other indoor |  | -3.00** |  | 3.92*** |
| Friends/classmates/colleagues, Other indoor | 2.72** |  |  |  |
| Multiple categories, At home |  |  |  | 2.83** |
| Note: *p <.05, **p <.01, ***p <.001, only variables p<0.05 are presented  IQR: interquartile range, HDS: healthy diet score, LPA: light physical activity, MVPA: Moderate to vigorous physical activity, PA: physical activity, PBDP: plant-based diet propensity score, SB: sedentary behavior | | | | |

**Supplementary Table S5**: Characteristics of participants according to input variables for clustering and multi-behavioral clusters (N= 510); the WEALTH study

| **Variable** | ***Unhealthy***  ***behavior*** | ***Mixed behavior – healthy diet*** | ***Mixed behavior – stable good mood*** | ***Healthy behavior*** |
| --- | --- | --- | --- | --- |
|  | N = 109 | N = 173 | N = 142 | N = 86 |
| **Sleep quality median** | 61.0 (50.0, 71.0) | 60.5 (49.0, 68.5) | 79.0 (66.0, 88.0) | 71.8 (58.5, 84.5) |
| **Sleep duration median** | 7.0 (6.2, 7.6) | 7.0 (6.5, 7.5) | 7.0 (6.6, 7.7) | 7.0 (6.4, 7.6) |
| **Fatigue** |  |  |  |  |
| Median | 41.0 (32.5, 49.5) | 42.5 (35.0, 52.5) | 20.0 (11.0, 26.0) | 27.0 (19.5, 37.0) |
| IQR | 20.5 (14.5, 27.5) | 21.0 (15.5, 27.0) | 14.1 (9.0, 21.0) | 20.0 (12.0, 31.0) |
| **Valence** |  |  |  |  |
| Median | 71.5 (63.5, 77.5) | 67.3 (60.5, 73.8) | 87.0 (82.0, 94.5) | 82.4 (75.0, 89.5) |
| IQR | 13.6 (9.9, 19.3) | 15.3 (10.0, 20.0) | 6.3 (4.0, 9.4) | 8.9 (6.5, 14.1) |
| **Calmness** |  |  |  |  |
| Median | 67.5 (61.0, 76.5) | 65.0 (55.0, 71.3) | 85.3 (79.0, 94.0) | 80.0 (72.5, 85.5) |
| IQR | 15.0 (9.8, 21.9) | 15.8 (10.5, 23.4) | 8.2 (4.4, 11.0) | 10.2 (7.0, 19.0) |
| **Energetic arousal** |  |  |  |  |
| Median | 57.3 (51.0, 67.5) | 56.8 (50.3, 63.5) | 80.8 (75.0, 88.5) | 73.9 (64.5, 82.0) |
| IQR | 22.5 (17.0, 29.9) | 20.9 (15.1, 27.4) | 13.1 (8.5, 21.0) | 18.4 (13.0, 27.3) |
| **PBDP (overall)** | 61.5 (55.8, 67.6) | 75.0 (66.9, 84.2) | 68.8 (60.2, 77.5) | 68.1 (61.5, 74.7) |
| **PBDP (healthy)** | 34.5 (27.3, 41.9) | 61.7 (50.9, 68.9) | 48.9 (39.1, 61.2) | 53.6 (42.6, 63.1) |
| **PBDP (unhealthy)** | 27.0 (21.7, 34.6) | 14.8 (10.3, 19.2) | 17.6 (11.0, 24.7) | 12.5 (9.2, 20.0) |
| **Healthy Dietary Score** | 21.0 (15.0, 27.0) | 32.0 (26.0, 38.0) | 30.0 (23.0, 36.0) | 34.0 (27.0, 39.0) |
| **Number of meals median** | 3.5 (3.0, 5.0) | 4.0 (3.0, 6.0) | 4.0 (3.0, 5.0) | 5.0 (4.0, 7.0) |
| **First meal timing median** | 9.5 (8.4, 10.6) | 8.8 (7.6, 9.6) | 8.4 (7.5, 9.5) | 8.1 (7.3, 8.7) |
| **Night-time fasting median** | 15.4 (13.2, 17.3) | 13.0 (11.8, 14.3) | 13.1 (11.7, 15.0) | 12.1 (11.2, 13.3) |
| **LPA** |  |  |  |  |
| Median | 143.3 (110.8, 162.8) | 118.2 (96.7, 139.8) | 122.2 (95.3, 144.8) | 184.6 (150.5, 217.0) |
| IQR | 60.8 (39.5, 85.8) | 35.7 (22.1, 49.8) | 38.3 (24.8, 58.0) | 49.6 (35.2, 72.5) |
| **MVPA** |  |  |  |  |
| Median | 76.8 (59.8, 94.3) | 79.3 (62.9, 95.5) | 78.7 (57.5, 93.8) | 98.7 (71.8, 126.6) |
| IQR | 39.5 (27.5, 52.4) | 32.5 (21.6, 49.2) | 33.3 (20.9, 43.3) | 34.4 (22.3, 54.5) |
| **Sedentary time** |  |  |  |  |
| Median | 583.3 (524.6, 620.8) | 564.7 (510.7, 636.2) | 583.9 (524.6, 630.1) | 452.0 (404.8, 492.4) |
| IQR | 131.5 (90.5, 179.0) | 115.8 (77.7, 151.8) | 120.0 (80.4, 169.0) | 110.1 (79.7, 161.2) |
| **Standing time** |  |  |  |  |
| Median | 200.1 (163.4, 239.7) | 223.7 (182.7, 271.6) | 214.6 (176.9, 262.9) | 325.1 (249.2, 375.6) |
| IQR | 87.2 (58.0, 117.8) | 62.2 (41.2, 85.4) | 66.8 (44.6, 92.8) | 77.8 (54.4, 113.3) |
| **Sitting time** |  |  |  |  |
| Median | 451.4 (382.9, 505.5) | 498.1 (448.4, 570.3) | 500.7 (443.7, 575.2) | 368.7 (308.5, 410.1) |
| IQR | 154.1 (106.7, 198.2) | 124.3 (92.0, 173.0) | 129.2 (85.7, 182.9) | 108.2 (84.0, 144.3) |
| **Number of sit to stand transitions** |  |  |  |  |
| Median | 47.0 (41.0, 58.0) | 52.0 (43.0, 60.0) | 46.5 (38.0, 55.0) | 55.5 (45.5, 68.0) |
| IQR | 12.5 (8.0, 16.5) | 10.0 (7.5, 15.5) | 10.0 (7.5, 13.0) | 13.3 (9.5, 21.0) |
| **Eating behavior context stability** | -2.1 (-2.4, -1.8) | -1.9 (-2.1, -1.5) | -1.8 (-2.1, -1.4) | -2.0 (-2.4, -1.7) |
| **PA context stability** | -3.0 (-3.3, -2.7) | -2.8 (-3.2, -2.4) | -2.9 (-3.2, -2.4) | -3.0 (-3.2, -2.6) |
| **SB context stability** | -2.3 (-2.6, -1.8) | -1.8 (-2.3, -1.3) | -1.8 (-2.3, -1.5) | -2.2 (-2.8, -1.7) |
| **Most frequent eating behavior context modality** |  |  |  |  |
| Alone, At home | 48.6 (53) | 52.0% (90) | 60.6% (86) | 33.7% (29) |
| Alone, At college/work | 3.7 (4) | 6.9% (12) | 2.8% (4) | 2.3% (2) |
| Alone, Other indoor | 2.8 (3) | 0.6% (1) | 0.0% (0) | 0.0% (0) |
| Family/children, At home | 21.1 (23) | 27.7% (48) | 25.4% (36) | 33.7% (29) |
| Family/children, In a green space | 0.0 (0) | 0.0% (0) | 0.0% (0) | 1.2% (1) |
| Family/children, On the move/street | 0.0 (0) | 0.0% (0) | 0.0% (0) | 0.0% (0) |
| Family/children, Other indoor | 4.6 (5) | 3.5% (6) | 2.8% (4) | 11.6% (10) |
| Friends/classmates/colleagues, At home | 4.6 (5) | 0.0% (0) | 0.7% (1) | 0.0% (0) |
| Friends/classmates/colleagues, At college/work | 3.7 (4) | 4.6% (8) | 1.4% (2) | 7.0% (6) |
| Friends/classmates/colleagues, In a green space | 0.0 (0) | 0.0% (0) | 0.0% (0) | 1.2% (1) |
| Friends/classmates/colleagues, On the move/street | 0.9 (1) | 0.0% (0) | 0.7% (1) | 0.0% (0) |
| Friends/classmates/colleagues, Other indoor | 7.3 (8) | 0.6% (1) | 1.4% (2) | 1.2% (1) |
| Multiple categories, At home | 1.8 (2) | 2.9% (5) | 4.2% (6) | 8.1% (7) |
| Multiple categories, In a green space | 0.0 (0) | 0.6% (1) | 0.0% (0) | 0.0% (0) |
| Multiple categories, Other indoor | 0.0 (0) | 0.0% (0) | 0.0% (0) | 0.0% (0) |
| Strangers/crowd/organized group/other, At college/work | 0.0 (0) | 0.6% (1) | 0.0% (0) | 0.0% (0) |
| Strangers/crowd/organized group/other, Other indoor | 0.9 (1) | 0.0% (0) | 0.0% (0) | 0.0% (0) |
| **Most frequent PA context modality** |  |  |  |  |
| Alone, At home | 13.8 (15) | 20.2% (35) | 18.3% (26) | 7.0% (6) |
| Alone, At college/work | 12.8 (14) | 6.9% (12) | 12.0% (17) | 5.8% (5) |
| Alone, In a green space | 2.8 (3) | 3.5% (6) | 2.8% (4) | 5.8% (5) |
| Alone, On the move/street | 17.4 (19) | 28.3% (49) | 20.4% (29) | 22.1% (19) |
| Alone, Other indoor | 11.0 (12) | 5.8% (10) | 8.5% (12) | 3.5% (3) |
| Alone, Other outdoor | 2.8 (3) | 1.2% (2) | 1.4% (2) | 8.1% (7) |
| Family/children, At home | 4.6 (5) | 2.9% (5) | 9.2% (13) | 7.0% (6) |
| Family/children, In a green space | 0.0 (0) | 0.0% (0) | 0.0% (0) | 3.5% (3) |
| Family/children, On the move/street | 2.8 (3) | 4.0% (7) | 1.4% (2) | 2.3% (2) |
| Family/children, Other indoor | 5.5 (6) | 3.5% (6) | 4.2% (6) | 7.0% (6) |
| Family/children, Other outdoor | 0.0 (0) | 0.0% (0) | 0.0% (0) | 1.2% (1) |
| Friends/classmates/colleagues, At home | 0.9 (1) | 0.6% (1) | 0.0% (0) | 0.0% (0) |
| Friends/classmates/colleagues, At college/work | 13.8 (15) | 0.6% (1) | 7.7% (11) | 7.0% (6) |
| Friends/classmates/colleagues, In a green space | 0.9 (1) | 0.0% (0) | 0.0% (0) | 2.3% (2) |
| Friends/classmates/colleagues, On the move/street | 1.8 (2) | 2.9% (5) | 0.7% (1) | 1.2% (1) |
| Friends/classmates/colleagues, Other indoor | 7.3 (8) | 2.3% (4) | 2.8% (4) | 2.3% (2) |
| Friends/classmates/colleagues, Other outdoor | 0.0 (0) | 0.6% (1) | 0.7% (1) | 3.5% (3) |
| Multiple categories, At home | 0.0 (0) | 0.0% (0) | 0.0% (0) | 2.3% (2) |
| Multiple categories, At college/work | 0.0 (0) | 0.0% (0) | 0.0% (0) | 1.2% (1) |
| Multiple categories, In a green space | 0.0 (0) | 0.6% (1) | 0.0% (0) | 0.0% (0) |
| Multiple categories, On the move/street | 0.0 (0) | 0.6% (1) | 0.0% (0) | 1.2% (1) |
| Multiple categories, Other indoor | 0.0 (0) | 0.6% (1) | 0.7% (1) | 0.0% (0) |
| Multiple categories, Other outdoor | 0.0 (0) | 0.6% (1) | 0.7% (1) | 2.3% (2) |
| Strangers/crowd/organized group/other, At home | 0.0 (0) | 0.0% (0) | 0.7% (1) | 0.0% (0) |
| Strangers/crowd/organized group/other, In a green space | 0.0 (0) | 0.0% (0) | 0.7% (1) | 0.0% (0) |
| Strangers/crowd/organized group/other, On the move/street | 1.8 (2) | 5.2% (9) | 1.4% (2) | 2.3% (2) |
| Strangers/crowd/organized group/other, Other indoor | 0.0 (0) | 9.2% (16) | 4.9% (7) | 1.2% (1) |
| Strangers/crowd/organized group/other, Other outdoor | 0.0 (0) | 0.0% (0) | 0.7% (1) | 0.0% (0) |
| **Most frequent SB context modality** |  |  |  |  |
| Alone, At home | 57.8 (63) | 56.6% (98) | 58.5% (83) | 39.5% (34) |
| Alone, At college/work | 7.3 (8) | 13.9% (24) | 15.5% (22) | 8.1% (7) |
| Alone, In a green space | 0.0 (0) | 0.0% (0) | 0.0% (0) | 0.0% (0) |
| Alone, On the move/street | 0.9 (1) | 0.0% (0) | 1.4% (2) | 0.0% (0) |
| Alone, Other indoor | 0.0 (0) | 1.7% (3) | 2.1% (3) | 3.5% (3) |
| Family/children, At home | 7.3 (8) | 11.0% (19) | 11.3% (16) | 18.6% (16) |
| Family/children, At college/work | 0.0 (0) | 0.0% (0) | 0.0% (0) | 0.0% (0) |
| Family/children, In a green space | 0.0 (0) | 0.0% (0) | 0.7% (1) | 0.0% (0) |
| Family/children, On the move/street | 0.0 (0) | 0.0% (0) | 0.0% (0) | 3.5% (3) |
| Family/children, Other indoor | 5.5 (6) | 0.6% (1) | 1.4% (2) | 12.8% (11) |
| Friends/classmates/colleagues, At home | 0.9 (1) | 1.2% (2) | 0.0% (0) | 0.0% (0) |
| Friends/classmates/colleagues, At college/work | 14.7 (16) | 12.7% (22) | 7.7% (11) | 5.8% (5) |
| Friends/classmates/colleagues, In a green space | 0.0 (0) | 0.0% (0) | 0.0% (0) | 1.2% (1) |
| Friends/classmates/colleagues, Other indoor | 4.6 (5) | 0.6% (1) | 0.0% (0) | 1.2% (1) |
| Multiple categories, At home | 0.0 (0) | 0.0% (0) | 0.0% (0) | 3.5% (3) |
| Multiple categories, At college/work | 0.9 (1) | 0.0% (0) | 0.0% (0) | 0.0% (0) |
| Multiple categories, Other indoor | 0.0 (0) | 0.0% (0) | 0.0% (0) | 0.0% (0) |
| Strangers/crowd/organized group/other, At home | 0.0 (0) | 0.0% (0) | 0.7% (1) | 0.0% (0) |
| Strangers/crowd/organized group/other, At college/work | 0.0 (0) | 0.6% (1) | 0.0% (0) | 0.0% (0) |
| Strangers/crowd/organized group/other, Other indoor | 0.0 (0) | 1.2% (2) | 0.7% (1) | 2.3% (2) |
| % (n) for categorical variables; Median (Q1, Q3) for continuous variables  IQR: interquartile range, HDS: healthy diet score, LPA: light physical activity, MVPA: Moderate to vigorous physical activity, PA: physical activity, PBDP: plant-based diet propensity score, SB: sedentary behavior | | | | |

**Participants who completed WEALTH protocol**

**N= 627**

**n= 116**

**Participants having at least one missing value among:**

Median sleep quality score: n=22; Median sleep duration: n=23

Fatigue: median n=2; IQR n=2; Valence: median n=2; IQR n=2;

Calmness: median n=2; IQR n=2; Energetic arousal: median n=2; IQR n=2

Overall PBDP: n=18; Healthy PBDP: n=18; Unhealthy PBDP: n=18; HDS: n=18

Meal frequency: n=8; First meal timing: n=16; Median night-time fasting: n=22

Context stability EB: n=11; Most frequent EB context modality: n=8

LPA: median n=1; IQR n=1; MVPA: median n=1; IQR n=1

Context stability PA: n=27; Most frequent PA context modality: n=15

Context stability SB: n=34; Most frequent SB context modality: n=16

Sedentary time: median n=35; IQR n=35; Standing time: median n=35; IQR n=35

Sitting time: median n=35; IQR n=35; Sit to stand transitions: median n=35; IQR n=35

**Participants with complete data on clustering input variables**

**N= 511**

**n= 1**

**Participants having missing value in occupation**

**Participants with complete data on all variables**

**N= 510**

**Supplementary Figure S1**: Flowchart of participants; IQR: interquartile range, HDS: healthy diet score, LPA: light physical activity, MVPA: Moderate to vigorous physical activity, PA: physical activity, PBDP: plant-based diet propensity score, SB: sedentary behavior

|  | Variance  percent | Cumulative variance  percent |
| --- | --- | --- |
| Dim 1 | 5.31 | 5.31 |
| Dim 2 | 4.02 | 9.33 |
| Dim 3 | 3.63 | 12.96 |

 

 

**Supplementary Figure S2**: Inertia and Mean Squared Error of Prediction (MSEP) values for Factor Analysis on Mixed Data

 

**Supplementary Figure S3**: Contribution of variables on principal components driven from Factor Analysis on Mixed data; red line represents p=0.05. IQR: interquartile range, HDS: healthy diet score, LPA: light physical activity, MVPA: moderate to vigorous physical activity, PA: physical activity, PBDP: plant-based diet propensity score, SB: sedentary behaviors

   

 

**Supplementary Figure S4**: Gain in inertia by number of clusters and individual representation on retained principal components by cluster
